# Correction: Reoccuring neural stem cell divisions in the adult zebrafish telencephalon are sufficient for the emergence of aggregated spatio-temporal patterns

**DOI:** 10.1371/journal.pbio.3001132

**Published:** 2021-02-17

**Authors:** Valerio Lupperger, Carsten Marr, Prisca Chapouton

The affiliations for the third author were listed incorrectly. The correct affiliations for Prisca Chapouton are:

3 Helmholtz Zentrum München–German Research Center for Environmental Health, Institute of Stem Cell Research, Neuherberg, Germany

4 Biomedical Center, Department for Cell Biology and Anatomy, Medical Faculty, Ludwig-Maximilians-Universität, Planegg-Martinsried, Germany
