# Supplementary figures and images for: Reoccurring neural stem cell divisions in the adult zebrafish telencephalon are sufficient for the emergence of aggregated spatiotemporal patterns
Source: PLoS Biol. 2020 Dec 8;18(12):e3000708. doi: 10.1371/journal.pbio.3000708 (PMC7748264; doi:10.1371/journal.pbio.3000708)

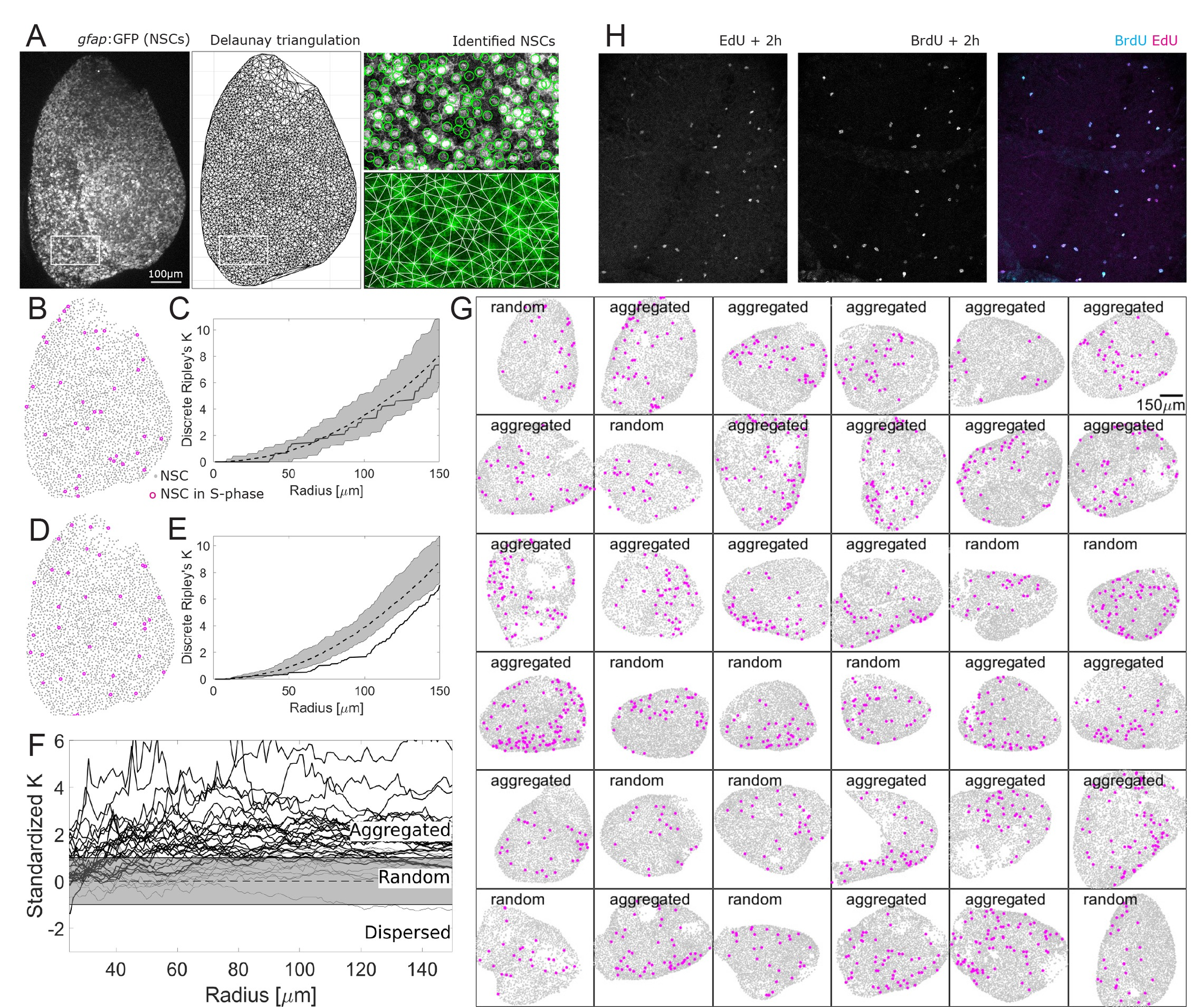

Supplement: S1 Fig — (A) Delaunay triangulation on the identified NSCs to calculate discrete Ripley’s K. (B) A synthetically generated random pattern of NSCs in S-phase. (C) Discrete Ripley’s K correctly identifies the pattern in (B) to be within the 90% CI of randomly sampled patterns. (D) A synthetically generated dispersed pattern with an interaction radius of 100 μm. (E) Discrete Ripley’s K correctly identifies the pattern in (D) below the random interval. (F) Standardized K shows S-phase NSC aggregation in 24 (thick lines) out of 36 hemispheres. Hemispheres that show a mean standardized K value higher than 1 between 30 and 150 μm are classified as aggregated (thick lines). Each trace represents the spatial distribution of S-phase NSCs in 1 hemisphere. (G) All hemispheres of the dataset. Gray dots represent NSCs magenta dots represent NSCs in S-phase. Aggregation patterns are classified according to the standardized K analysis in (F). (H) Cells in S-phase are equally labeled by EdU or BrdU when those are administered concomitantly. BrdU, 5-bromo-2′-deoxyuridine; CI, confidence interval; EdU, 5-Ethynyl-2′-deoxyuridine; NSC, neural stem cell. (TIF) [file pbio.3000708.s001.tif]

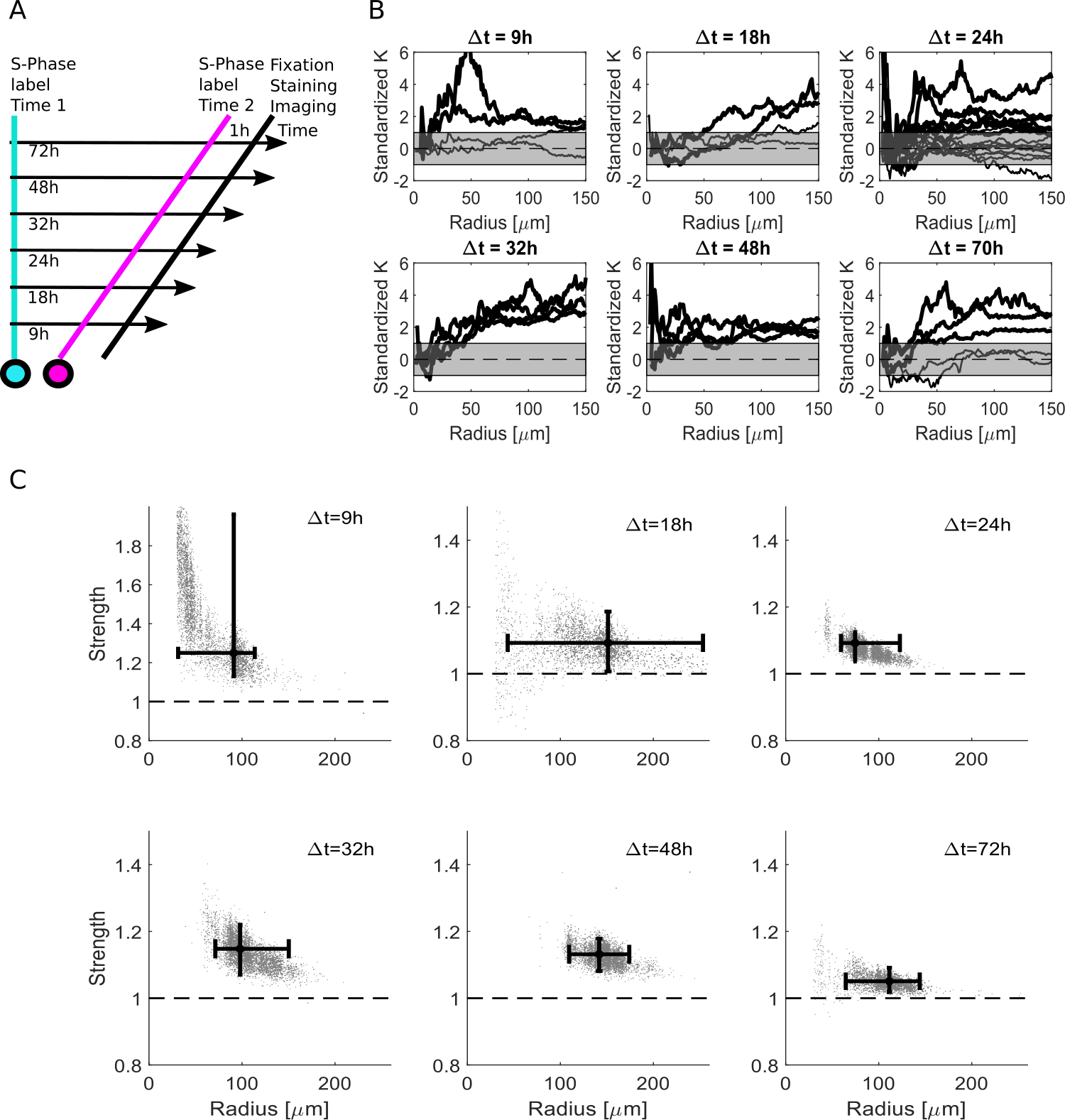

Supplement: S2 Fig — (A) Experimental setup for measuring spatiotemporal patterns. With different labeling intervals Δt between the 2 S-phase labelings, we systematically profile spatiotemporal effects. (B) Standardized K shows spatiotemporal aggregation of S-phase NSCs in 22 out of 36 hemispheres for different Δt, while the remaining 14 patterns are classified as random. (C) Parameter inference on all labeling intervals Δt. Most likely interaction strength and radius for all hemispheres per labeling interval determined via posterior sampling density. Whiskers cover the 95% CIs for strength and radius. CI, confidence interval; NSC, neural stem cell. (TIF) [file pbio.3000708.s002.tif]

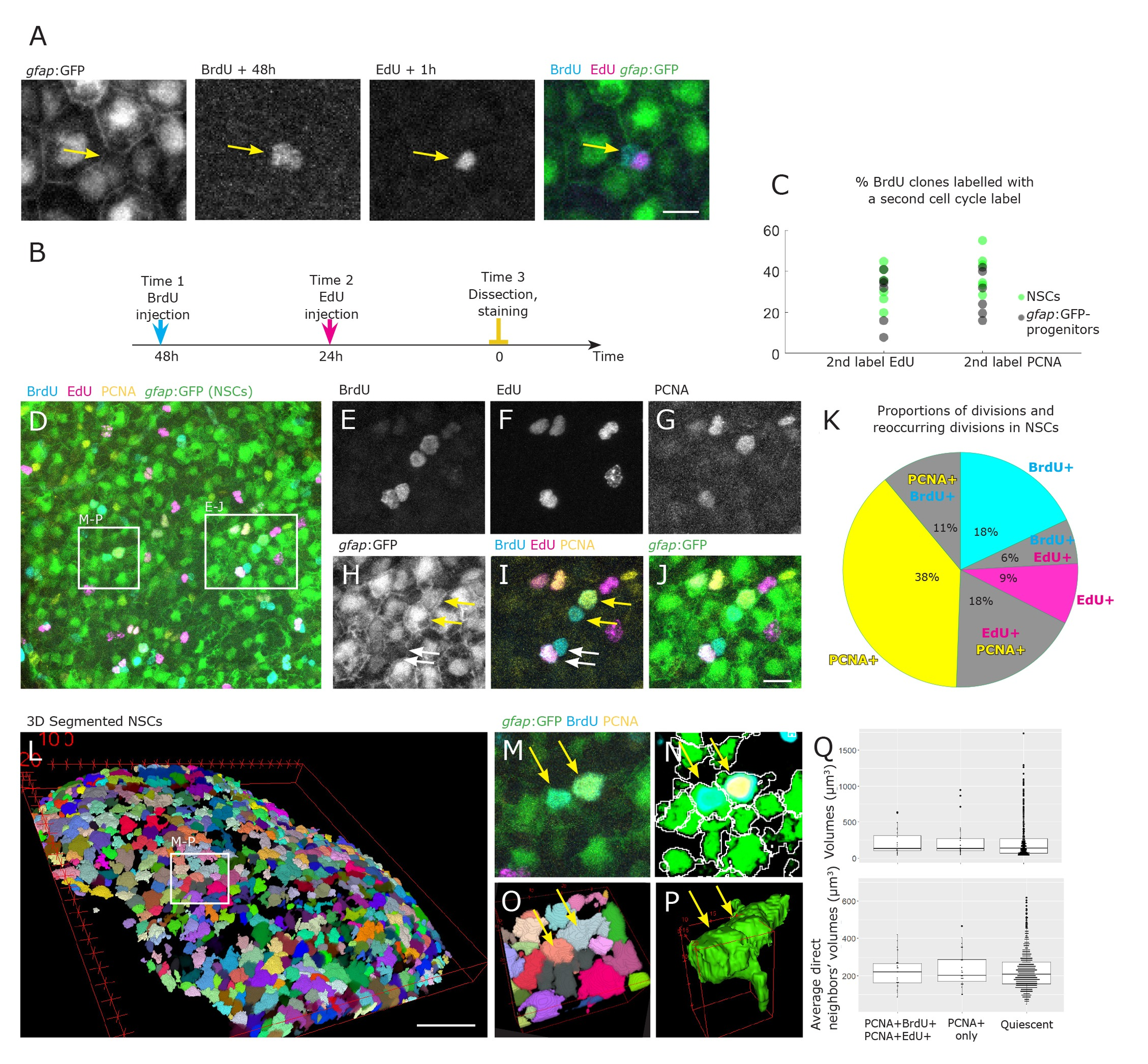

Supplement: S3 Fig — (A) Example of reoccurring divisions taking place in gfap:GFP− progenitors (yellow arrow). (B) Experimental setup for observing NSCs in S-phase at 3 time points within 2 days. (C) Reoccurring divisions have been quantified in 6 hemispheres out of 4 brains (% of BrdU+clones labeled with a second cell cycle label) and are observed both in NSCs (gfap:GFP+, green dots) and in gfap:GFP− progenitors (gray dots) after 1 day (BrdU+EdU+) or after 2 days (BrdU+PCNA+). Higher percentages of redivisions are observed compared to the experiments with a short pulse of EdU labeling at time 2 (injection 1 h before killing, see Fig 3), probably due to more S-phases entries labeled with a longer duration of EdU availability in this experimental setup. (D-J) Maximum intensity projection and close-up view of a 4-channel image depicting recurrent divisions marked with 3 cell cycle labelings at 3 time points: 2 days earlier by BrdU (E), 1 day earlier by EdU (F), and at the time of fixation by PCNA (G). (H) gfap:GFP+ daughter cell pairs (green arrows) and gfap:GFP− daughter cell pairs (white arrows) with respectively 1 daughter entering a second round of division. (I,J) Overlays. Scale bar: 10 μm. (K) Quantification of reoccurring divisions in NSCs performed on 63× magnification images from 4 hemispheres. The average proportions of dividing NSCs labeled at distinct time points reveal that about 40% of all PCNA+ NSCs have been generated by a recent division (compare gray quadrants PCNA+EdU+ and BrdU+EdU+ to the yellow PCNA-only quadrant) (L) 3D segmentation and 3D visualization of all NSCs performed on the same image as shown in (D–I), representing a total of 834 NSCs. (M–P) Close-up view of the boxed area delineated in D and L depicting BrdU-labeled sister cells, 1 of which is labeled with PCNA (yellow arrows). (M) Maximum intensity projection. (N) Single z-plane. All NSCs are delimited by white lines obtained by a 3D watershed segmentation applied after Ilastik pixel classification of the [file pbio.3000708.s003.tif]

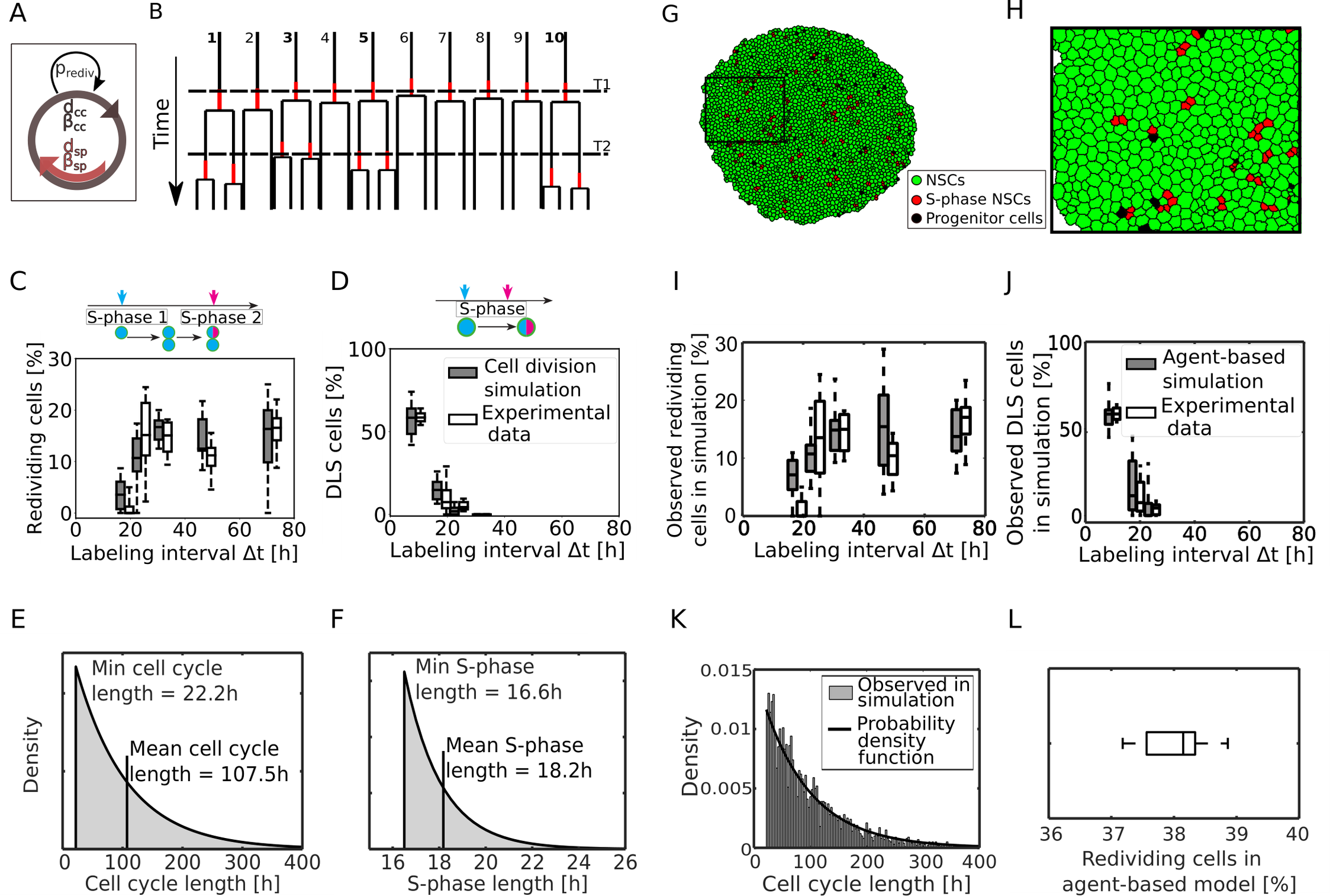

Supplement: S4 Fig — (A) We devise a cell division model to describe dividing NSCs with 5 parameters: the minimal cell cycle length dcc and minimal S-phase length dsp and their variability βcc and βsp, respectively, parametrizing a lag-exponential distribution and the redivision probability prediv. Note that we do not have any information about the length of G2 and thus do neither model nor visualize this phase in the graphs. (B) We use observed redivision proportions (see Fig 3T) and the percentage of DLS cells (see Fig 3F) to feed a division model (S-phases labeled in red) and take simulated measurements at T1 and T2 to infer the redivision probability pre-div. We observe a difference between the model (prediv = 0.38) and observed redivisions frequency (0.15): From 10 simulated cells dividing at T1, 4 cells (cell 1, 3, 5, and 10, shown in bold) redivide. Thus, 40% cell redivide, which is close to pre-div. However, our measurement at T2 only picks up 2 cells (cells 3 and 5) corresponding to only 20%, in line with the observed redivision frequency. (C, D) The cell division model fits the proportion of redividing cells (C) and DLS cells (D) for different labeling intervals. (E) We find an extremely variable delayed exponential distributed cell cycle with a minimal length (dcc) of 22.2 h and a mean (dcc + βcc) of 107.5 h. (F) The S-phase length is narrow with 16.6-h minimal length (dsp) and βsp = 1.5h. (G) Snapshot of an agent-based simulation using the Morpheus software corresponding to Fig 4A (Time point 2) with NSCs (green), NSCs in S-phase (red), and dividing intermediate progenitor cells (black). (H) Boxed area in (G) shown in higher magnification to visualize simulated cell shapes. (I, J) Proportion of observed redividing and DLS cells for different labeling intervals in agent-based simulations vs. experimental data. (K) Cell cycle length measured in the agent-based simulations compared to the probability density function of the estimated delayed exponential function in (E). (L) Fra [file pbio.3000708.s004.tif]
