# Supplementary material for: Reoccurring neural stem cell divisions in the adult zebrafish telencephalon are sufficient for the emergence of aggregated spatiotemporal patterns
Source: PLoS Biol. 2020 Dec 8;18(12):e3000708. doi: 10.1371/journal.pbio.3000708 (PMC7748264; doi:10.1371/journal.pbio.3000708)
Supplement: Attachment — Submitted filename: Review-PBIOLOGY-D-20-00516R1.pdf [file pbio.3000708.s007.pdf]

Review of the manuscript entitled "Aggregated spatio-temporal division patterns emerge from reoccurring divisions of neural stem cells" by Lupperger et al.

This manuscript tackles the issue whether the cell cycle in neural stem cells (NSC) is controlled at the supercellular level or if these cells divide in a stochastic cell intrinsic manner. They use as case study the NSC of the zebrafish teleencephalon. They characterise the spatio-temporal patterns of nucleoside analogue multi-labelling in a large number of cells and tissues (80000 cells in 36 brains). They first reveal, using simple statistics, an non-uniform aggregation pattern of the labelled cells. They then go on to model the spatial patterns in two steps. They first estimate the cell cycle and S-phase length distribution parameters, assuming that both follow delay-exponential density functions, neglecting the spatial information in the data set. They reveal that about 15% of the daughter cells re-enter cell cycle corresponding to an intrinsic probability per cell of 0.38. Taking the parameters of the distributions from this first step, as cell intrinsic parameters, they implement a spatial simulation that can reproduce some of the statistical features of the spatial-temporal patterns. They conclude that the concordance of the simulations and the observed patterns indicates that cell intrinsic control of the cell cycle and cell cycle reentry maybe sufficient to explain the observed spatio-temporal patterns of cell division. They disfavour the alternative hypothesis of an aggregating mechanisms caused by populational interactions and niches.

The question of the cell intrinsic versus extrinsic control of stem cell dynamics and spatio-temporal tissue patterning is important and timeless. The authors approach leveraging experimental data, statistics and computational modelling is clever and insightful. The article can be improved from its present form taking into account several points.

My major issues with the manuscript are the following:

1. The estimates of the cell cycle and S-phase lengths seem to be awfully long. The S-phase length with minimal time dsp=16h and mean dsp+betasp=18.2h seems especially long. The coefficient of variation of betasp/(dsp+betasp)≈10% also indicates that the cells were extremely precise in controlling this time. In practice this means that NSCs would spend at least 16h replicating DNA and would then complete the process within few hours. This is at odds with 8-9h length and coefficient of variation 20-40% reported by Weber et al. (2014) in cell lines of lymphocyte lineage, or the 6h minimum minimum and average of 10-12h in cell lines using FUCCI as reported by Grant et al. (2018; doi:10.1080/15384101.2018.1547001). This may reflect a cell type or tissue. Alexiades & Cepko (1996) reported a large progressive deceleration of cell cycle with mean S-phase length of 5h at E14 and up to 18h at PO during development of rat retina, with S-phase remaining about 40% of total cell cycle. However, more recent studies seem to indicate shorter S-phases. Point et al. (2013; doi:10.1073/pnas.1219563110) reported estimates for mean S-phase length in stem cells (4h), neuroblasts (9h) and intermediate progenitors (14-17h) of mammaliam brain that were much lower than the estimates here. Also, Arai et al. (2011; doi:10.1038/ncomms1155) report values for S-phase length of 3-5 h. These values are

very difficult to reconcile with the minimal absolute value of the S-phase of 16h. These may reflect biases in the estimates or biologically relevant differences between cell lineages or animals that must be addressed.

2. On page 11 the formula for f(x;beta,d) is incorrect. The function f is should be a probability density function of the cell cycle or S-phase length x. Yet, the formula presented is clearly not a probability density function. First, its integration in the range x in [0,+Infinity] does not yield 1. Second, it is inconsistent as it sums d, with dimensions time (e.g. hour), with a normalised exponential distribution that has no dimensions. I think the authors meant to write something like:

$$f(x;\beta,d) = \begin{cases} 0 & x < 0 \\ \frac{1}{\beta} e^{-\frac{1}{\beta}(x-d)} & x \geq d \end{cases}$$

Alternatively, they may use a single equation with the Heaviside function as in the equation 1 of Weber et al (2014). Whether this was a simple typo or reflects the way the simulations were parameterised and performed in framework Morpheus must be re-examined by the authors.

3. The authors use a clever strategy to estimate the parameters of the their model of stochastic cell cycle entry and reentry based on intrinsic delayed-exponential distributed total cell cycle and S-phase lengths. They first estimate the four parameters of the two distributions ignoring all spatial constraints and then perform a spatial simulation using the framework Morpheus. This simulation has some additional ingredients, including the fixed division rate pdiv, the removal of “differentiated” cells after one division, the minimum and maximal cell size, and all the inherent parameters of the simulation. It was unclear how these additional ingredients affect final statistics of the cell cycle division in the simulations. What I mean is: did the authors reanalyse the results of the spatial simulations in the same way that they analysed the labelling data to make sure that effective cell cycle times are the same? The additional ingredients namely the pdiv could in principle offset the effective cell cycle times in the simulations. There is nothing in the description of the simulations that ensures that this was not the case. Such biases could explain the difference in the interaction strength and radius.

Other lesser issues:

4. The authors say that they detect *“a* <u>*non-random*</u>*, aggregated patterns of dividing NSCs in the zebrafish brain.”* The statement *“non-random“* is inadequate and perpetuates the “common sense” notion that random equates to uniform probability (in this case uniform in space). The authors mean non-uniform or non-isotropic or …. The truth is they themselves later characterise the cell cycle and S-phase lengths, the cell-cycle reentry, the differentiation as random processes.

5. Page 4. *“cells in S-phase straddling the Deltat and incorporating both labels”*. Does this mean that the cells spent more than Deltat in S-phase replicating their DNA ? How can the authors distinguish if the cells where all the time in S-phase or were undergoing one S-phase during the first labelling and S-phase from a subsequent round of division during second labelling ?

6. The authors' argument is one of parsimony. They argue that there is no need to evoke niches and supercellular interactions to explain the aggregation patterns because cell intrinsic rates of cell cycle entry and re-entry (re-division) would be sufficient to explain the patterns. Yet, this parsimony is more a modelling one than a biological one. What are the molecular mechanisms underpinning the rate of cell cycle re-entry or re-division? The authors make a fair discussion on the question of "how these recurrent divisions of adult stem cells come about". However, they may wish to acknowledge that it may be equally plausible mechanistically to explain the local reentry in cycle as a consequence of random foci of production of cell cycle progression signals (as mentioned in point 7 below).

7. The modelling result that omitting intrinsic cell-cycle re-entry leads to random uniform spatial patterns is not general. It is specific to the simulations performed and based on the constant rate that the cells divide. Once the cells are place according to a uniform distribution in space the constant rate results in a "random" spatial patterns. The lack of intrinsic re-division may not be incompatible with spatio-temporal aggregation pattern. Most likely if there would be transient foci of production of a growth factor necessary for overcoming G1 arrest (for example) one would see "random" cell division in space (as the foci would uniformly distributed in space) but with an apparent aggregation pattern and even apparent cell-cycle re-entry if the transient foci lasted long enough.

8. The repeated use of the term "analogon" surprised me. I didn't recall reading or hearing it. I am used to "analog" or "analogue". Googling "analogon" brought mostly dictionaries and thesaurus related to German or Dutch. There are only 5 pages of papers using "analogon" in pubmed versus, about 5000 using "analog" or "analogue". These precedents of using "analogon" notwithstanding, maybe the authors should consider adopting the more conventional term.

9. Section "*A positive interaction model fits…"* (page 4). It would help the reader if the authors would explain the rational and interpretation of the "interaction strength". The reference to Lupperberger et al. and the formal definition in the corresponding methods section is not sufficient to allow the reader to make sense of *"we find that a model with an interaction radius of ~ 100 µm and an interaction strength > 1 describes the data best"*. See also the next point.

10. Fig. 3B. The interaction strength and radius seem not to be independent of each other, showing a conspicuous negative correlation. Why is that? How does it affect the result?Furthermore, the posterior distribution seems to be multimodal with some periodicity as a function of the radius. This might be an optical illusion but similar patterns appear in supplementary fig. 3. Is this an effect of the ABC sampling? Is the most "most likely radius" of 93µm the point of higher probability density?

11. Page 10. "*We also observed re-divisions in gfap: GFP- cells, which were clearly distinguishable from each gfap: GFP+ cells (Supplementary Figure 4A) and occured in similar proportions (Supplementary Figure 4J).*" This sentence is confusing and should be better explained. Does this mean that NSC (GFP+) and other cells (GFP) divide with the same temporal patterns?

12. The authors report the uncertainty of their data with a 2 significant figure e.g. 14.4±8.1% on page 10. Although reporting of data is increasingly relaxed in the scientific literature it is worth recalling the rule that the reported estimated values should be rounded to the first decimal of the uncertainty.

13. Page 10, second paragraph starting with *"To confirm our observations,…"*. Are the results consistent with a stationary process and balanced growth of the tissue ? Are the statistics on the first (L1) to second (L2) labelling the same as for the second to third (L3) labelling? What I envisage is a 4 by two contingency table of the type: rows L1-L2-, L1+L2-,L1-L2+,L1+L2+ vs row first set L1L2:BrdU-24h-EdU and second set L1L2:EdU-24h-PCNA. Are the values in these tables the same? The similarity could be addressed using Chi2 test.

14. Page 6. The authors use incorrectly the term *"predicted"* to refer to *"estimated"* values, as in the sentence *"It predicted a minimal cell cycle time of 22.2h".*

15. The authors denoted by beta the mean of the exponential component of the delayed-exponential cell cycle length distribution. This is a different notation from the one used in the reference they cite (Weber et al., 2014). In Weber et al. alpha and beta are respectively the mean of the exponential and the delay of the delayed-exponential. There is nothing wrong with adopting a new notation (beyond the faulty equation mentioned above). To avoid confusing the reader, however, may be the authors should make a brief note about this.

16. The authors estimated a probability of cell cycle re-entry of 0.38, which is, on face value, not very different from 0.5. Is the value 0.5 within the confidence or credibility interval of the estimate? Would the results be very different if the authors would impose a value of 0.5? Intriguingly, a value of 0.5 could be interpreted as asymmetric cell division which is one of the classical models for stem cell self-renewal and differentiation. Maybe the authors would like to address this issue.

17. Maybe the authors would like to discuss their results in the context of the article by Mura et al. (2019; doi: 10.1371/journal.pcbi.1007054).
