# Supplementary material for: Reoccurring neural stem cell divisions in the adult zebrafish telencephalon are sufficient for the emergence of aggregated spatiotemporal patterns
Source: PLoS Biol. 2020 Dec 8;18(12):e3000708. doi: 10.1371/journal.pbio.3000708 (PMC7748264; doi:10.1371/journal.pbio.3000708)
Supplement: Attachment — Submitted filename: Lupperger et al. PLoS Bio Response.pdf [file pbio.3000708.s008.pdf]

Reviewer 1

This manuscript analyses the distribution of dividing adult telencephalic neural stem cells in the adult zebrafish brain. They determine that the distribution is weakly clustered (non-random) and by analysing divisions at two time points they find the locations of the second divisions are significantly correlated to the location of the first division. Some stem cells go through two successive divisions in a relatively short time. The authors interpret their data as evidence that a stem cell's recent history of division influences the probability of that stem cell dividing again. The authors favour a cell intrinsic mechanism for this phenomenon rather than local niche environment, but there is no evidence either way. I feel this work is still at the descriptive stage, somewhat preliminary and doesn't yet offer much insight into the biology of the topic.

Some things I would like to know:

If you compare the distribution of divisions across all brains is there a pattern in their locations or is it completely random? From Figure 3 it looks like more divisions at the periphery of the territory they analyse? And relatedly, are any of the division clusters related to known regional territories within the telencephalon?

We thank the reviewer for raising these interesting questions, which we would like to address one by one.

- Following the reviewer's suggestion, we analyzed if more cells divide at the periphery of the analyzed brain regions. To that end, we first identified border cells as those cells that span the convex hull of triangulation. In each hemisphere *i*, we then determined the distance of all S-phase NSCs S_i to the next border cell. Finally, we compared the distribution of these distances to the distribution of distances from S_i randomly sampled NSCs. We find that in only 8 out of 36 (22%) hemispheres the distance distribution is significantly different from random ($p < 0.05$, KS test). We repeated the analysis for redivisions and found only 1 out of 24 (4%) hemispheres to be significantly different ($p < 0.05$). Thus, we do not believe that divisions occur more frequently at the periphery of the hemispheres in a systematic way. Rather, we find that the aggregations are located in areas that vary from hemisphere to hemisphere, a fact that is hopefully now more comprehensible due our newly added Supplementary Fig. 1G where we show all 36 division patterns.
- To investigate whether there are regional territory-related patterns, we manually annotated and analyzed lateral pallium (Dl), medial pallium (Dm), and anterior pallium (Da) in 25 hemispheres. In accordance with Dray et al. (Development 2015), we observe a higher proportion of S-phase NSCs in the Dm (see figure below, left). Interestingly, the proportion of re-divisions is similar in all regional territories (see figure below, right).

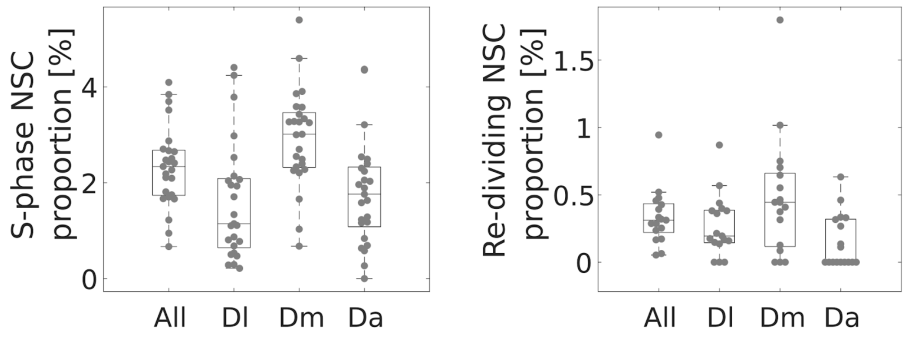

- We next evaluated the regional territories in the same way as done for the whole hemispheres (compare Supplementary Figure 1F) and applied the Ripley's K statistics separately. We discarded regions with less than 10 S-phase NSCs due to statistical power, ending up with 35 regions from 25 hemispheres. We found that all regions show either aggregated or random, but no dispersed patterns. The proportion of aggregated patterns declined from 48% (12/25) in Dm over 42% (3/7) in Dl, to 33% (1/3) in Da.

We would thus like to note that (i) separating hemispheres into smaller subregions reduces the number of divisions and thus impairs our overall statistical power, and (ii) we often observe patterns that span regional territories (see exemplary hemisphere right). This encourages us in our approach to perform hemisphere-wide pattern analyses. We now discuss the option to separate hemispheres into regional territories in the revised version of our manuscript:

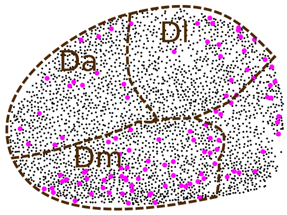

“Following distinctions in the division activity described previously in Dray et al. (2015), we compared division proportions and spatial patterns in three different hemisphere domains (lateral, medial, anterior) and found that these domains also show aggregated and random, but no dispersed patterns. Since separate domains contain in ~40% of all cases less then 10 S-phase NSCs and patterns often span across domains, we refrain from analyzing them separately.”

Why do the authors favour a cell intrinsic mechanism to explain the non-random distribution rather than local niche environment?

We agree with the reviewer that a role played by a local niche environment in driving the patterns of division cannot be rejected. We develop and address this aspect in two paragraphs in our revised Discussion, where we now write:

“According to this model, coordination of cell cycle entries in the population can be explained by the internal synchronization due to the cells' history. Such a model is supported by interesting studies following cell families in cultured cell lines, detecting correlations of cell cycle parameters between siblings and cousin cells as a result of inherited factors but independent on their location (Mura et al., 2019; Sandler et al., 2015). This cell-intrinsic driven behavior is in contrast to cellular systems endowed with a clear environmental regulation, in particular in stem cell compartments constituted by a distinctive 3D architecture like in the bone marrow or the hair follicle in the skin. There, the precise location of stem cells is associated with their cycling behavior and fate (Rompolas et al., 2013), indicating that the position within an environment impacts on the stem cells activity. In the neurogenic zone studied here, no distinctive organization of the tissue would so far hint towards morphological features specifically associated with a NSC's activity. Our analysis of cellular volumes in this study does not indicate that a distinctive density dependent niche would be in place. In this context, it is also interesting to note that two intermingled types of progenitors with radial morphology in the killifish telencephalon reveal distinct proliferative behaviors (Coolen et al., 2020) even if sharing the same environment, arguing for a small contribution of environmental effects on stem cell activities.

Nevertheless, we cannot reject the existence of cell extrinsic mechanisms that might contribute to the spatio-temporal patterns, such as local diffusive signaling activity in delimited groups of cells, a functional activity of extended cell-cell contacts (as has been observed in NSCs by Obermann et al. (2019)), or the activity of the Notch signalling pathway (Chapouton et al., 2010). As a result of either mechanisms, levels of molecular heterogeneity have been observed: For instance, variable levels of the Zinc finger protein Fezf2 regulate Notch activity levels and quiescence of NSCs (Berberoglu et al., 2014). Likewise, the expression of miR9 involved in keeping quiescence upstream of Notch signaling is found only in a subset of quiescent cells (Katz et al., 2016) several days after a division. And in a recent study, a subpopulation of NSCs that expresses low levels of Elavl3 has been characterized as mostly non-dividing cells in transit towards neuronal differentiation (Lange et al., 2020).”

Following the reviewers comment, we analyzed whether niches would be morphologically detectable in the system studied here, measured the volumes of all NSCs and compared the dividing NSCs and their direct

neighbors (see our newly added Supplementary Figure 3L-Q). The results do not indicate that a density dependent niche would be in place.

What do the authors think might be the advantage to the non-random distribution?

The consequences of the aggregated patterns might be an addition of new born neurons in close-by proximity during specific time windows, allowing for neuronal circuits between newborn neurons to be built. We added this speculation at the very end of our revised Discussion, where we now write:

"Hence, in stem cell populations, individual phases of quiescence and exit thereof might well be predictable according to inherited factors, allowing us to understand how the kinetics of tissue maintenance are regulated. Beyond this study, which aimed at understanding a regulation within days-scale time window, long term tracing studies will help understanding the consequences of those patterns on the organisation of the resulting neuronal circuits."

Reviewer 2

The manuscript by Lupperger et al. with the title: "Aggregated spatiotemporal division patterns emerge from reoccurring divisions of neural stem cells" describes the analysis and mathematical modeling of a comprehensive dataset on patterns of adult neural stem divisions using whole mounts of the zebrafish telencephalon. Most of the stem cells in the zebrafish telencephalon are quiescent. The divisions occur in aggregated, non random patterns. A significant proportion of daughter stem cells reenter the cell cycle. Modeling the cycling behavior suggests that this re-entry into S-phase is a key determinant of the observed patterns of stem cell divisions. The authors conclude that a cell's cycling history is an important parameter in the spatiotemporal aggregation of dividing stem cells regardless of possible feedback mechanisms in the population. All together, this work addresses the important issue ie that of the balance between proliferation and quiescence of stem cells.

The strength of this study lies in the fact that it is quantitative at a large scale and uses simulation as a tool to reveal new insights. I am in favour of publishing this paper. There are a few problems that need to be solved before. To assure a broad readership, the authors should make a bigger effort to bridge the gap between the disciplines. For mostly traditionally trained cell biologist the explanation of approaches and models needs to be improved.

Specifically:

I find the introduction and the description of the experiments extremely dense. What is for example the difference between dispersed and random and aggregated and non-random. Please provide graphical models in the supplement illustrating the principle differences between the different arrangements.

We thank the reviewer for this instructive comment. We now added a Box on “Statistical analysis of spatio-temporal point patterns”, where we define the terms random, aggregated, and dispersed and put them into the wider context of point pattern analysis. We also revised the introduction and the description of experiments. Moreover, following the reviewer’s suggestion, we added Supplementary Figures 1B and 1D where we show how simulated random and dispersed patterns look like. Finally, we added Supplementary Figure 1G, in which we show all observed spatial division patterns together with their label (aggregated vs. random). We hope these additions make our approach and the tools applied better understandable.

For classification of patterns we added a short paragraph in the revised Methods:

“To classify S-phase patterns as aggregated, random or dispersed we calculated the mean z-score between 30 and 150 µm. If the mean z-score is above 1 we classify the pattern as aggregated, between -1 and 1 as random and below -1 as dispersed.”

Are these aggregates the same as previously determined regions of higher proliferation activity?

The aggregates observed here are distinct from the previously described subdomains of the telencephalon, the medial (Dm), anterior (Da) and lateral (Dl) domains (described in Dray et al., 2015). We have carefully considered this aspect and found that all regions show either aggregated or random patterns, but no dispersed patterns. The proportion of aggregated patterns declined from 48% (12/25) in Dm over 42% (3/7) in Dl, over to 33% (1/3) in Da. We often observed patterns that span regional territories and chose to perform our analyses on the entire hemisphere in order to maintain a high statistical power in the pattern analyses. We comment on these regional territories in the revised Results, where we now write:

“Since separate domains contain in ~40% of all cases less then 10 S-phase NSCs and patterns often span across domains, we refrain from analyzing them separately.”

The telencephalon also contains a ventral region with a strong concentration of PSA-NCAM expressing dividing progenitors. This region however was not contained in our imaged z-stacks.

I am not sure whether Fig. 1B should be included in the main body. In any case it needs, guidance to anatomical details. Add an arrow to indicate telencephalon for example.

Following the reviewer's suggestion, we discarded this panel in the revised version of our manuscript.

Page 10 When they refer to the frequency of aggregated division patterns they observe this only for 70% of the cases. How do the cases look where this aggregation is not evident?

Following the reviewer's remark, we now visualize all 36 spatial division patterns analyzed and the classification according to Ripley's K statistic in Supplementary Figure 1G (see also below). We would like to note that the randomness of a division pattern (pink points) can only be assessed while taking the set of possible division locations (gray points) as a substrate into account. Here, our analysis deviates from the standard spatial statistics as described e.g. in the book of Baddeley et al. (2015). We clarify this aspect of our analysis in the newly added Box.

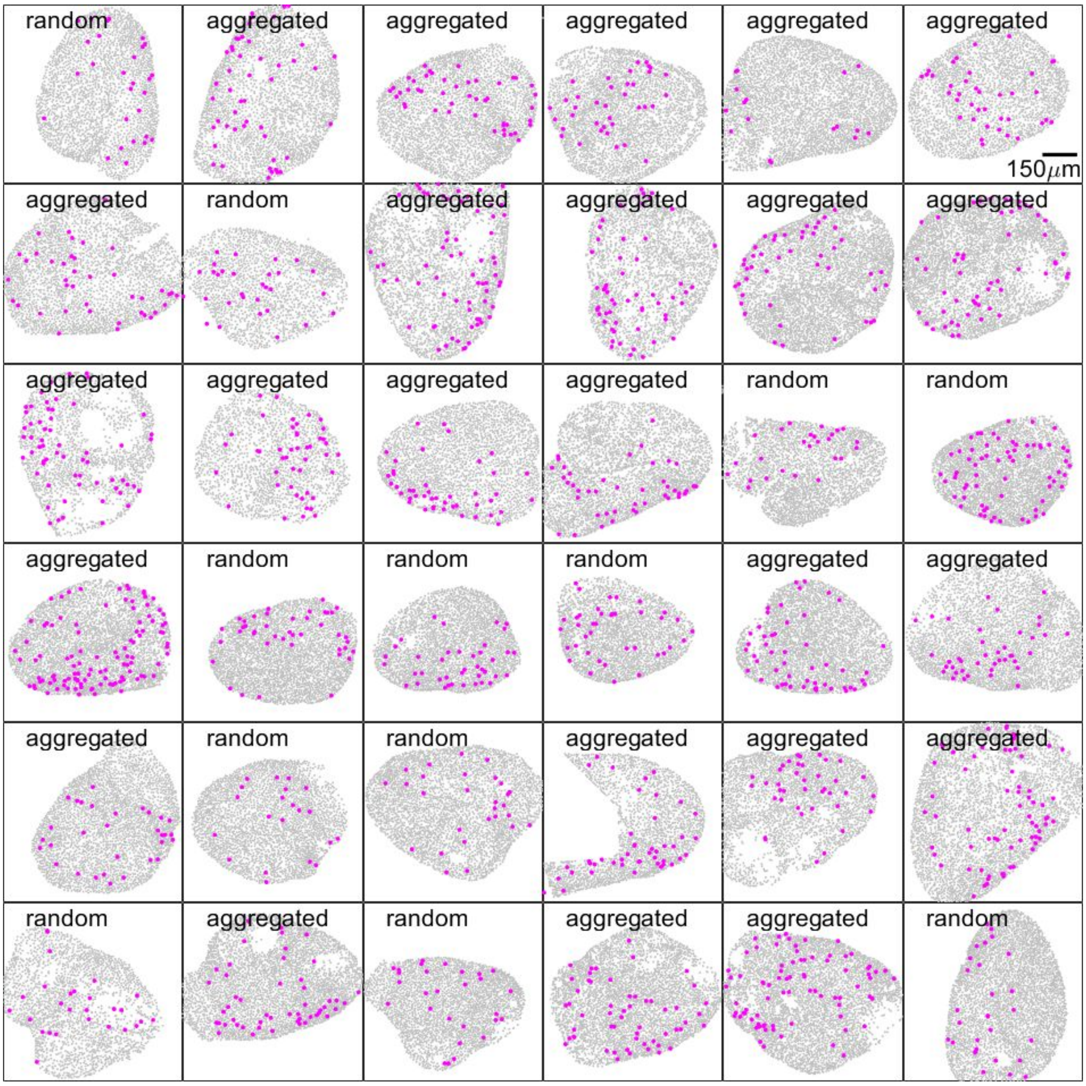

Why is this the case? Is this a reflection of the biology of the experimental assessment of the patterns.

We tried to address this question in the revised Discussion of our manuscript, where we now write:

"The quantification of S-phase NSC patterns is challenged by their heterogeneity and the relatively weak signal: Only 70% of the 36 hemispheres show aggregated spatial patterns, the remaining 30% qualify as random, so we wondered how this heterogeneity comes about. Since we estimate NSCs to re-divide with only 38% probability, we believe that de-novo divisions of previously quiescent cells dilute the aggregation patterns arising from reocurring divisions."

What are the quality standards? Manual verification is mentioned. But what is the result of this?

To ensure a high quality of single cell identification, we checked every automatically identified S-phase NSC by manually examining its S-phase label (EdU, BrdU) and gfap:GFP intensity as they are the basis of our analysis. We tried to clarify the respective descriptions in our revised manuscript where we now write:

"We identified the 3D coordinates of *gfap*:GFP+ NSCs (Figure 1C,D,D') and of EdU+ nuclei (Figure 1C'-D) automatically. To analyze the spatial pattern of S-phases, we used the coordinates of all *gfap*:GFP+ NSCs (Figure 1D') as a reference grid (see Supplementary Figure 1A). Via manual inspection we then discriminated between EdU+*gfap*:GFP+ cells, representing NSCs in S-phase, and EdU+*gfap*:GFP- cells, representing intermediate progenitors in S-phase (März et al., 2010) (Figure 1D'',E)."

How similar are the patterns of divisions between different animals?

To address this interesting question we analyzed parameter correlations in brains where we had both left and right hemispheres and compared those to randomly sampled hemisphere pairs. We found that both strength and radius show a weak positive correlation for the 11 hemisphere pairs (see Figure below). Next, we permuted the values and calculated correlations again. Doing this n=10000 times allows us to calculate empirical p-values for radius and strength correlation. We find that the empirical p-value is neither significant for the radius (p=0.12), nor for the strength (p=0.25). We thus conclude that patterns between animals are similarly uncorrelated as patterns between pairs of hemispheres.

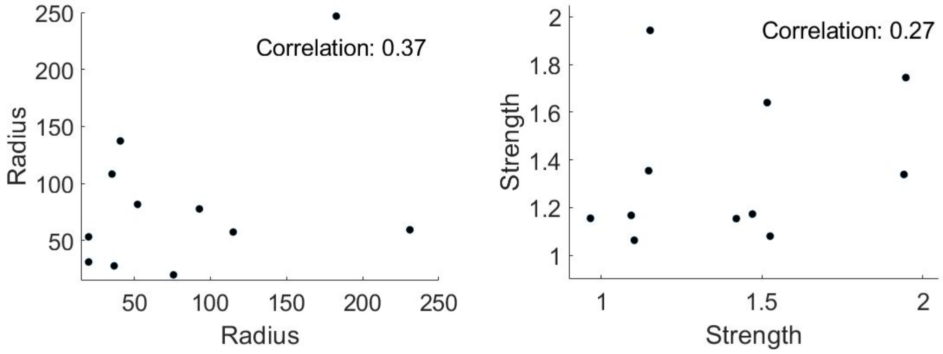

The patterns of division appear non-random in space ie some regions are more proliferating than others. I guess this is what you refer to as aggregated? It would thus not be a surprise that in these regions cells within a certain radius will re-enter cell cycle more frequently than anywhere else.

We define the term 'aggregated' more carefully in the revised version of the Results, where we write in the newly added Box:

"Non-random patterns can be classified into two types: When events are closer to each other than expected from the random null model, we call the pattern aggregated. If events are further apart from each other, we call the pattern dispersed."

We agree that re-entering the cell cycle is one of the options that come to mind when observing aggregated patterns. However, it is still necessary to detect and quantify those patterns carefully in a spatio-temporal manner. We revised the paragraph in the Results, where we motivate the analysis of cell cycle re-entry:

“Our analysis revealed a weak aggregation of successive NSCs in S-phase. Aggregated spatio-temporal patterns can emerge for several reasons: Signalling waves that stimulate cells to divide in a particular region, activation via cell-cell contacts, or a behavior specified by the particular state of a cell. We thus inquired whether a NSC’s history would be of relevance for new S-phase entries and made use of the double-labelling approach introduced above.”

Moreover, the GFAP-gfp positive cell bodies do not appear to be equally distributed but rather follow the pattern of dividing cells. Is this not a trivial result - the more NSCs in a region the more frequent cell cycle entry in this region?

The reviewer raises a very valid point here, but we already account for the possible inhomogeneous NSC distribution across the hemispheres when analyzing S-phase NSC patterns: To simulate random null model patterns for the Ripley’s K analysis we only sample from NSC locations. I.e. regions with a higher NSC density will also in the null model lead to regions with higher S-phase NSC density. To better explain our approach, we now write in the Methods:

“As the underlying NSC distribution could be inhomogeneous we adapted the measure to account for this possible inhomogeneity by sampling a point pattern only from discrete NSC locations and thus call it discrete Ripley’s K.”

"To investigate whether proliferative activity regulates new S-phase entries, we analyzed spatial division patterns at different time points." I fail to see the evidence for regulation. It may be correlation. But the specific layout of the niche itself may provide the necessary positive cues to trigger cell cycle re-entry. Thus, re-division would then not be caused by the cell intrinsic history of divisions.

We agree with the reviewer that regulation is the wrong term here. We use correlation and regulation more carefully in our revised manuscript and replaced the respective sentence with:

“To investigate whether proliferative activity correlates with new S-phase entries in a spatio-temporal manner, we made use of a second thymidine analog, BrdU, and observed consecutive S-phases taking place in vivo.”

Regulation of cell cycle re-entry might have an extrinsic component and we do not rule out that a niche effect could arise around an active NSC. We showed in fact in a previous study that Notch signaling is activated in neighbors of recently divided cells (Chapouton etal., J. Neurosci. 2010). To analyze whether niches would be morphologically detectable we added a 3D volumetric analysis and found no deviation in cell size between NSCs in S-phase and quiescent NSCs. The corresponding, newly added paragraph in our revised Results read:

“To assess whether cellular niches might be involved in the observed reoccuring divisions e.g. via the formation of groups of NSCs with distinctive volumes, we segmented single NSCs in 3D whole mount brain images of 4 hemispheres and measure their volumes (see Methods and Supplementary Figure 3L-P). We considered three groups of NSCs: (i) PCNA+ dividing NSCs, (ii) PCNA+BrdU+ and PCNA+EdU+ re-dividing NSCs and (ii) non-dividing NSCs without any marker. Measuring the volumes of all these NSCs and all immediately touching neighbors did not reveal any significant difference between the three groups (Kruskal-Wallis test, p-values = 0.1 and 0.37 respectively, Supplementary Figure 3Q). Hence, the NSC volume does not reveal any distinctive organisation around actively dividing NSCs, arguing against a model where cell density dependent niches would be associated with NSC activity.”

What does "A positive interaction model fits the observed spatio-temporal patterns" mean for the biology of stem cells. Does this mean that a dividing cell is influencing a second cell to divide positively in a 100 micron radius.

We use an interaction model to infer parameters describing the type (aggregated vs. random vs. dispersed) and extend (the range or radius) of the observed patterns, in line with standard spatial statistics using e.g. the Gibbs model. This however does not imply that the patterns are generated by an interaction process. We tried to explain our approach by revising the corresponding paragraph in the Discussion of our revised manuscript:

“The emergence of complex patterns from simple rules has been analyzed extensively, e.g. for artificial systems (Marr and Hütt, 2005), biology inspired models (Kauffman and Clayton, 2006), and biological phenomena (Manukyan et al., 2017). Here, we first use an interaction model to determine the strength and the radius of the observed pattern of S-phase NSCs. This extends traditional Gibbs or Cox models (Baddeley et al., 2015) to the analysis of a particular multi-type spatial point pattern where S-phase NSCs appear at discrete locations defined by the presence of all NSCs. However, fitting an interaction model to spatio-temporal data does not necessarily imply that cell-cell interactions are present. We thus model quiescent and S-phase NSCs as agents in a continuous space with stochastic cell cycle kinetics to quantitatively compare two hypotheses: Are extrinsic effects like cell-cell interactions or signalling waves required to generate the observed aggregated spatio-temporal division patterns, or do re-divisions of NSCs suffice? We find that agent-based simulations with re-dividing NSCs suffice to explain the spatio-temporal patterns observed in the zebrafish brain.”

How many stem cells are in this area?

We added this information to the text where we now write:

“On average, we find 202 ± 63 NSCs (mean ± std.dev. from n=36 brains) and 6 ± 4 NSCs in S-phase in a 100µm radius around an NSC in S-phase.”

Can one obtain really evidence for interaction from these models and data? The conclusion from the agent based spatio-temporal model is different. I find this confusing.

We agree with the reviewer that our description of the two models should be clearer. In general, fitting an interaction model to spatio-temporal data does not necessarily imply that cell-cell interactions are present (see above). Instead, we find evidence that re-divisions suffice to generate similar patterns in silico. However, if re-divisions rely on interactions with neighboring cells or not, we cannot resolve with our approach. We hope that the revised paragraph in the Discussion (see above) helps to convey our thoughts on that.

"To confirm our observations,….." In this and the following paragraph on page 5, I lack the statistical analysis of the statements. These need to be included as above in the text.

We thank the reviewer for that comment. For this set of experiments we have now performed additional countings and calculated the proportions of reoccuring divisions: 43 ± 4% of PCNA+ NSCs are marked with a second label while 21 ± 7% of EdU-labelled NSCs are labelled by BrdU. These results are presented in Supplementary Table 3 and in Supplementary Figure 3K. This set of experiments shows that the use of a third cell cycle label also reveals cell cycle re-entries of NSCs, as in the first set of experiments.

We changed the term “confirm” and now write: “To support these results […]”

"To quantitatively evaluate if re-divisions suffice to induce the observed aggregated patterns, we simulated dividing NSCs with an agent based spatio-temporal model."

Please do make an effort and give an explanation what this approach is and what the benefits are for an agent based spatio-temporal model.

Following the reviewer's request, we now describe our approach in more detail in the revised Results section of our manuscript:

"Such a model simulates the actions and interactions of autonomous agents, in our case NSCs. Here, every single cell can be modeled at every time point while in other non-agent-based approaches one only gets summary statistics or averages per time point."

How do the patterns of division correlate with single cell sequencing data. Do these data provide markers for the NSCs prone to divide again?

Indeed, single cell sequencing of the zebrafish telencephalon and a thorough analysis of progenitors clusters has been performed by Cosacak et al. (Cell Report, 2019.) combining control and amyloidβ treated animals. There, the authors identified several clusters of progenitor cells and distinctive cell cycle transcripts signatures, of which some might correspond to the NSCs that divide several times, even though such events are proportionally very seldom and might be diluted in the dataset. A pseudotime analysis suggested a continuum of progenitor states. A direct comparison with our analysis and a precise spatial mapping of the distinct groups of progenitors is nevertheless difficult.

We have added this aspect in our Discussion:

"Single cell sequencing data, such as performed by Cosacak et al. (2019) might help associating these events with specific molecular pathways."

Reviewer #3:

The manuscript entitled "Aggregated spatio-temporal division patterns emerge from reoccurring divisions of neural stem cells" by Lupperger, Marr and Chapouton (PBIOLOGY-D-20-00516R1) address a problem of aggregation of cycling stem cells in the zebrafish telencephalon. The authors investigate if neural stem cells transiting through the cell cycle S-phase are coordinated in space and time within the population of the adult zebrafish telencephalon. By using a combination of the Ripley's test and a previously published mathematical model (Lupperger et al., 2018. Cytometry A. 93(3):314-322), the authors demonstrated that neural cells transiting through S-phase are non-random but weakly aggregated, both in space and time. By using dual labelling in S-phase, the authors inferred cell cycle and S-phase length of the telencephalon neural stem cells and observed a rapid cell cycle re-entry in about 15 % of the newly born stem cells. The authors developed a simple non-spatial probabilistic mathematical model which they fitted to their experimental data on the rapid cell cycle re-entry to then use their best-fitted parameter values in a more complex spatial agent-based model. The combined models predict that the presence of cell cycle re-entry reproduces the Ripley's test results of aggregation whilst its absence leads to random spatio-temporal distribution of cells transiting through S-phase. The authors concluded that "spatio-temporal aggregation of dividing stem cells can thus emerge from the cell's history, regardless of possible feedback mechanisms in the cell population".

The problem of how neural stem cells regulate cycling in the neural tissue is of great importance in developmental biology and its repercussions can be naturally allocated in neuroscience and other more applied areas of medicine. Hence, the problem is very interesting for a variety of readers of PLOS Biology and the authors addressed it by using innovative methods. The more original aspect of the study, in my opinion, is the use of Ripley's statistics, a statistical method well known in other areas of science, to determine spatial aggregation. Although the authors previously studied aggregation of neural stem cell division in the same tissue in a previous publication (Lupperger et al., 2018. Cytometry A. 93(3):314-322), they did not use this statistical method in the previous publication. As a matter of fact, they anticipated that they would use this method in the end of the discussion section of the previous paper. The next original aspect of the study is that the authors combined the popular BrdU/EdU dual labeling with Ripley's statistics to now calculate aggregation of cells marked with both labels. Since both labels are not only separated in space, but more importantly in time, this allowed them to evaluate spatio-temporal aggregation of cells. Thanks to these two aspects the authors highlight a non-trivial spatio-temporal pattern of cells transiting through S-phase. I believe that both, the methodology (novel in developmental biology, to my knowledge) and the result itself are more than interesting. Nevertheless, there are a few points regarding the modelling results and the conclusions from it that need to be addressed.

Major points:

1. The title of the manuscript has to reflect the specific findings of the study. The authors did not demonstrate that the aggregated spatio-temporal division patterns emerge from reoccurring divisions of neural stem cells, as the title of the submitted manuscript stays. Instead, they i) elegantly demonstrated the existence of an aggregated spatio-temporal division pattern, ii) they showed that a modelling approach incorporating cells re-entering into the cell cycle is sufficient to reproduce the aggregated pattern, iii) their model predicts that in the absence of cell cycle re-entering the aggregated pattern vanishes and iv) the study was performed in the adult zebrafish telencephalon. Hence, the manuscript title should reflect these four facts. Regarding the term "divisions", please see my Major comment Number 4.

We thank the reviewer for this suggestion and changed the title to:

“Agent-based modeling reveals that reoccuring neural stem cell divisions in the adult zebrafish telencephalon are sufficient for the emergence of aggregated spatio-temporal patterns”

We hope that we thus cover the 4 points listed by the reviewer while keeping the title understandable.

2. The interaction radius predicted from the agent-based model is about half of the experimentally determined. Why is that? Could it be due to the different geometry of the in silico tissue compared with the telencephalon hemispheres? The first one is 2D and circular whilst the second one is 3D and not precisely circular. To test this hypothesis the authors could test other geometries more similar to the experimentally tested.

We thank the reviewer for this suggestion. We use a cellular Potts model (Graner and Glazier 1992) implemented in Morpheus, which creates cell shapes that are actually very similar to cell shapes observed in the z-projection of the experimental data. We missed to show this in our original submission and now include an exemplary simulation snapshot plus zoom-in in Supplementary Figure 4G,H (see also below). Unfortunately, the Morpheus software only allows for simpler shapes (e.g. hexagons or squares) which we deemed inadequate for our simulations.

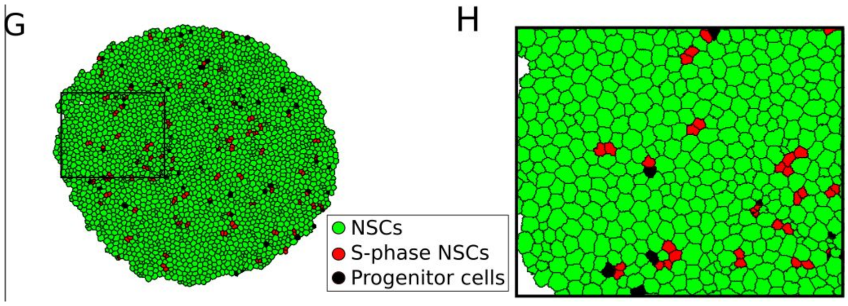

Concerning the deviations of the inferred radius in data vs. simulations, we extended our Discussion in the revised version of our manuscript, where we now detail limitations of our approach:

"Our agent-based simulations are able to reproduce aggregated patterns and their frequency, however with a smaller interaction radius then observed. Contributions to this deviation might come from the abstracted morphology of the hemisphere and individual cell shapes (see Supplementary Figure 4G,H), the discrepancy between a 2D model and 3D effects in the brain, or the previously reported underestimation of radii when using an Euclidean distance measure (Lupperger et al., 2018)."

3. In the second section of Results, the authors performed a spatio-temporal distribution Ripley's K analysis of the experiments in which the labelling interval was 32 hours. Then, they broadened the range of labelling intervals from 9 hours to 72 hours. They quantitatively identified 22 out 36 hemispheres spatio-temporal aggregated patterns of S-phase cells between the two labelling time points. What is the explanation of this heterogeneity? What happens with the other 14 hemispheres (nearly 40%)? Could this heterogeneity be explained with the models?

We thank the reviewer to bring up these important points. We extended the caption of Supplementary Fig. 2B accordingly, where we now write:

"Quantitatively, we identify in 22 out of 36 hemispheres spatio-temporally aggregated patterns of divisions between two labelling time points, while the remaining 14 patterns are classified as random. (see Supplementary Figure 2B)."

Following the reviewer's question further, we checked the heterogeneity in our simulated spatio-temporal data set and report the result in a revised paragraph in our manuscript:

“We could also reproduce the observed heterogeneity in the emergence of the spatio-temporal patterns: In 36 simulated hemispheres, we found 58 ± 8% aggregated patterns (n=10 independent simulations, mean ± std.) in accordance with our experimental observations (61%, see Supplementary Fig. 2B). Also in the simulations, the remaining patterns are classified as random throughout.”

One explanation of the observed spatial (and spatio-temporal) heterogeneity is now discussed in the revised Discussion:

“The quantification of S-phase NSC patterns is challenged by their heterogeneity and the relatively weak signal: Only 70% of the 36 hemispheres show aggregated spatial patterns, the remaining 30% qualify as random, so we wondered how this heterogeneity comes about. Since we estimate NSCs to re-divide with only 38% probability, we believe that de-novo divisions of previously quiescent cells dilute the aggregation patterns arising from reocurring divisions.”

4. Along the text, the authors describe cell divisions. But the authors do not actually measure cell divisions. They measure cells transiting through S-phase. Of course, these are cycling cells that eventually will divide. But this is not what they measured. Maybe they also measured cells in mitosis but I could not find in the manuscript any reference to that. The only cases in which the authors could state that the cells did divide is when the daughter cells entered in a second S-phase. Therefore, the authors should write 'cells transiting through S-phase' or 'cells in S-phase' or something of the sort instead of 'dividing cells'.

We thank the reviewer for that remark. We would like to note that we describe the appearance of doublet in the Methods where we write:

“In experiments with $\Delta t>18h$, the majority of Time 1-labelled cells were found as doublets, i.e. a pair of small cells close to each other (see Figure 4K,O,S), since the timespan allowed the mother cell to reach its mitotic (M) phase.”

Because nuclei labelled by BrdU or EdU appear as doublets 24 hours after incorporation, they most likely have been transiting through a mitotic event. We therefore allowed ourselves the use of the term “division”. Moreover, we revised the Results section, where we now write:

“Remarkably, we also noticed cells with both S-phase markers that, unlike DLS, were arranged as doublets, i.e. two daughter cells close to each other. These cells apparently entered a second S-phase, appeared in labelling intervals of Δt=24h, 32h, 48h, and 72h (Figure 3G-T), and were denoted as re-divisions accordingly. Such doublets allows us to extrapolate from the incorporation of S-phase labels to actual cell division events.”

5. In the second paragraph of the discussion section, the authors describe what they learn with their modelling approach. They state that " [we] quantitatively compare two hypotheses: Do we need cell-cell interactions or niche effects to generate the observed aggregated spatio-temporal division patterns, or do re-divisions of stem cells suffice? We find that the simplest model, i.e. one with no feedback but active, re-dividing NSCs is able to explain the emergent patterns observed in the zebrafish brains." I partially agree with this conclusion. I agree that they showed that their model, which does not account for feedback, or any other mechanism for that matter, is sufficient to reproduce their results. But I find two problems with the rest of the argument.

1) They did not quantitatively compare the performance of a model with feedbacks, or any other mechanism, with neither their model nor with the experimental data; hence, the sentence is not accurate.

We agree with the reviewer’s comment and revised the statement accordingly. We now write:

“We thus model quiescent and S-phase NSCs as agents in a continuous space with stochastic cell cycle kinetics to quantitatively compare two hypotheses: Are extrinsic effects like cell-cell interactions or signalling waves required to generate the observed aggregated spatio-temporal division patterns, or do re-divisions of NSCs suffice? We find that agent-based simulations with re-dividing NSCs suffice to explain the spatio-temporal patterns observed in the zebrafish brain.”

2) To term their model the simplest one because it lacks feedbacks, or any other mechanism, is at least debatable. I find many possible definitions of 'simplicity' and not all of them coincide with the one stated in the paragraph. Of course I understand what the authors imply but I consider that the concept of "simplicity" in this context is to vague and should be avoided. Overall, my conclusion is that that this paragraph should be rewritten.

We agree with the reviewer that our argumentation lacked stringency and needed reformulation. We extended and revised the Discussion accordingly, more carefully introducing different hypothesis and explicitly discussing the limitations of our approach:

“According to this model, coordination of cell cycle entries in the population can be explained by the internal synchronization due to the cells’ history. Such a model is supported by interesting studies following cell families in cultured cell lines, detecting correlations of cell cycle parameters between siblings and cousin cells as a result of inherited factors but independent on their location (Mura et al., 2019; Sandler et al., 2015). This cell-intrinsic driven behavior is in contrast to cellular systems endowed with a clear environmental regulation, in particular in stem cell compartments constituted by a distinctive 3D architecture like in the bone marrow or the hair follicle in the skin. There, the precise location of stem cells is associated with their cycling behavior and fate (Rompolas et al., 2013), indicating that the position within an environment impacts on the stem cells activity. In the neurogenic zone studied here, no distinctive organization of the tissue would so far hint towards morphological features specifically associated with a NSC’s activity. Our analysis of cellular volumes in this study does not indicate that a distinctive density dependent niche would be in place. In this context, it is also interesting to note that two intermingled types of progenitors with radial morphology in the killifish telencephalon reveal distinct proliferative behaviors (Coolen et al., 2020) even if sharing the same environment, arguing for a small contribution of environmental effects on stem cell activities.

Nevertheless, we cannot reject the existence of cell extrinsic mechanisms that might contribute to the spatio-temporal patterns, such as local diffusive signaling activity in delimited groups of cells, a functional activity of extended cell-cell contacts (as has been observed in NSCs by Obermann et al. (2019)), or the activity of the Notch signalling pathway (Chapouton et al., 2010). As a result of either mechanisms, levels of molecular heterogeneity have been observed: For instance, variable levels of the Zinc finger protein Fezf2 regulate Notch activity levels and quiescence of NSCs (Berberoglu et al., 2014). Likewise, the expression of miR9 involved in keeping quiescence upstream of Notch signaling is found only in a subset of quiescent cells (Katz et al., 2016) several days after a division. And in a recent study, a subpopulation of NSCs that expresses low levels of Elavl3 has been characterized as mostly non-dividing cells in transit towards neuronal differentiation (Lange et al., 2020).”

Minor points:

1. In section "A positive interaction model fits the observed spatio-temporal patterns", the "positive interaction" is not properly defined in the main text. The model characteristics are better described in the Methods section, subsection "Model based analysis", sub-sub section "Influence model" and more details can be obtained in the previous publication of the authors above mentioned. But, the nature of the interaction and what "positive" means should be properly described in the main text.

We agree that the term “positive” is wrong here, since also the interaction strength is per definition > 0. We tried to better describe our interaction model in the revised version of our manuscript where we now write:

"Ripley's K statistics is limited: It does not allow integrating different datasets, and it cannot quantitatively infer the strength and range of an observed pattern. To remedy these aspects, we use the temporal extension of a spatial model (Lupperger et al., 2018) that allows inferring the most likely parameters for interaction strength and interaction radius for an arbitrary number of datasets. Aggregated patterns emerge for an interaction strength > 1, random patterns for strength = 1, and dispersed patterns for strength < 1 (see Methods)."

2. In Figure 4U, the authors perform a two-sample Kolmogorov-Smirnov test comparing the neural stem cells that are in S-phase at a given time point with those re-entering S-phase. Nevertheless, a key assumption of the KS test is that the two samples are mutually independent. Unfortunately, the set of the second sample is included in the set of the first one. Hence, unless I miss something, they are not strictly independent.

The reviewer is correct that the two sets are not strictly independent and we are grateful for raising this point. We revised our analysis and now randomly sample divisions twice on each hemisphere to count the amount of appearing redivisions according to this null model. The figure is updated accordingly (see below) and we now write:

"While 14 ± 8% of NSCs in S-phase re-enter S-phase, only 1.9 ± 1.7% of randomly drawn divisions (same amount as observed per hemisphere) would be redrawn at random again ($p=9.4 \cdot 10^{-10}$, two-sample Kolmogorov-Smirnov test, Figure 3U)."

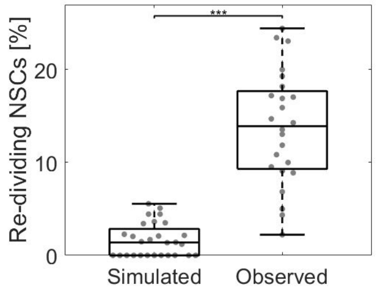

3. The authors feed the spatial model with the parameter values obtained from the simpler non-spatial model fitted to the re-cycling experimental data. These parameters are the minimal cell cycle length d_cc, the minimal S-phase length d_sp, their variability beta_cc and beta_sp and the re-division probability p_re-div. Did the authors verify that they properly work on the spatial model? In other words, did they verify that minimal cell cycle length and the minimal S-phase in the spatial model are what they should be? If they imposed a probability of re-division of 0.38. Did they empirically verify that the observed frequency of re-divisions is about 38 %? And so on... If the authors did these controls, they should be in the supplementary information.

Thanks for this suggestion. The added sub-figure (see below) will allow the reader to see that the estimated parameters are reproducible. We now show in Supplement Figure 4I-L that we are able to reproduce the estimated observed re-division and DLS fractions as well as the cell cycle length. Also the internal re-division fraction was confirmed with 38+-0.5%. S-phase lengths are by default correct as we just draw them from the respective distribution for the post-simulation analysis. We now write in the Results:

"Finally, we analyse whether the agent-based simulations are able to reproduce the input parameters, i.e. observed DLS and re-division fractions, cell cycle length and redivision probability. Re-division fractions and DLS fractions from simulations are similar to the ones observed in experimental data (Supplementary Figure 4I,J) ,and cell cycle lengths observed in the simulations fit nicely to the probability density function of the lag-exponential distribution (Supplementary Figure 4K). S-phase lengths are correct by default as we draw

them after simulation for post-simulation analysis, from the respective lag-exponential distribution. The proportion of re-dividing cells in the simulation is confirmed with 38 ± 0.5 % (Supplementary Figure 4L).”

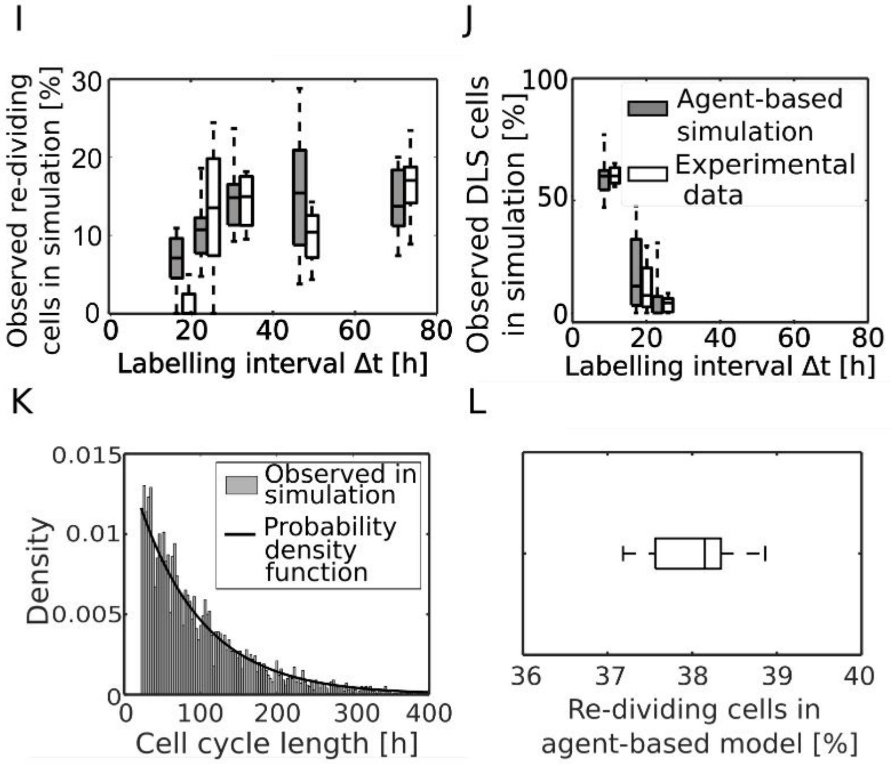

4. In the last paragraph of the Results section the authors described control simulations in which all the neural stem cells entered division with a probability of 2 x10(-3) per hour. First, how this value was obtained? Second, a probability by definition is unit-less, I guess what they are reporting is a rate. They should express it as such.

We thank the reviewer for pointing this out, changed “probability” to “rate” accordingly, and now report the estimation of the value in the Methods:

“We approximate a base division rate p_{div} from the average observed S-phase cell fraction (1.9 ± 0.7%, n=36 hemispheres, mean ± std) and S-phase length estimation (~18h): p_{div} = 0.019 /18h = $1•10^{-3}$ divisions per hour. This base division rate suffices for control simulations but for simulations with re-divisions we decreased p_{div} slightly. To obtain a similar amount of observed divisions as in the control simulations we fixed p_{div} = $9•10^{-4}$ divisions per hour to account for re-divisions, which divide after one cell cycle independently of the base division rate with probability $p_{re\text{-}div}$ = 0.38 (inferred from the cell division model above).”

We also realized that there was a typo in our initial manuscript ($2•10^{-3}$ instead of $1•10^{-3}$) and corrected that.

5. In the Figure 1 K, the discrete Ripley's curve displayed corresponds to the only one hemisphere observed in panel D and J, right? If so, this should be explicitly stated on the Figure legend.

The reviewer is correct. In the caption we now write:

“Discrete Ripley´s K quantification of the pattern shown in (E) reveals that NSCs in S-phase (solid line) are aggregated, that is, closer to each other than expected from random (dotted line with 90% confidence interval in grey) and dispersed patterns.”

Reviewer #4:

This manuscript tackles the issue whether the cell cycle in neural stem cells (NSC) is controlled at the supercellular level or if these cells divide in a stochastic cell intrinsic manner. They use as case study the NSC of the zebrafish telencephalon. They characterise the spatio-temporal patterns of nucleoside analogue multi- labelling in a large number of cells and tissues (80000 cells in 36 brains). They first reveal, using simple statistics, an non-uniform aggregation pattern of the labelled cells. They then go on to model the spatial patterns in two steps. They first estimate the cell cycle and S-phase length distribution parameters, assuming that both follow delay-exponential density functions, neglecting the spatial information in the data set. They reveal that about 15% of the daughter cells re-enter cell cycle corresponding to an intrinsic probability per cell of 0.38. Taking the parameters of the distributions from this first step, as cell intrinsic parameters, they implement a spatial simulation that can reproduce some of the statistical features of the spatial-temporal patterns. They conclude that the concordance of the simulations and the observed patterns indicates that cell intrinsic control of the cell cycle and cell cycle reentry maybe sufficient to explain the observed spatio-temporal patterns of cell division. They disfavour the alternative hypothesis of an aggregating mechanisms caused by populational interactions and niches.

The question of the cell intrinsic versus extrinsic control of stem cell dynamics and spatio-temporal tissue patterning is important and timeless. The authors approach leveraging experimental data, statistics and computational modelling is clever and insightful. The article can be improved from its present form taking into account several points.

My major issues with the manuscript are the following:

1. The estimates of the cell cycle and S-phase lengths seem to be awfully long. The S-phase length with minimal time dsp=16h and mean dsp+betasp=18.2h seems especially long. The coefficient of variation of betasp/(dsp+betasp)≈10% also indicates that the cells were extremely precise in controlling this time. In practice this means that NSCs would spend at least 16h replicating DNA and would then complete the process within few hours. This is at odds with 8-9h length and coefficient of variation 20-40% reported by Weber et al. (2014) in cell lines of lymphocyte lineage, or the 6h minimum and average of 10-12h in cell lines using FUCCI as reported by Grant et al. (2018; doi:10.1080/15384101.2018.1547001). This may reflect a cell type or tissue. Alexiades & Cepko (1996) reported a large progressive deceleration of cell cycle with mean S- phase length of 5h at E14 and up to 18h at PO during development of rat retina, with S-phase remaining about 40% of total cell cycle. However, more recent studies seem to indicate shorter S-phases. Point et al. (2013; doi:10.1073/pnas.1219563110) reported estimates for mean S-phase length in stem cells (4h), neuroblasts (9h) and intermediate progenitors (14-17h) of mammalian brain that were much lower than the estimates here. Also, Arai et al. (2011; doi:10.1038/ncomms1155) report values for S-phase length of 3-5 h. These values are very difficult to reconcile with the minimal absolute value of the S-phase of 16h. These may reflect biases in the estimates or biologically relevant differences between cell lineages or animals that must be addressed.

We thank the reviewer for this collection of relevant literature on S-phase measurements. We added a corresponding paragraph to the Discussion of our revised manuscript, where we now write:

“A cell division model allowed us to estimate cell cycle and S-phase length distributions from double labelling data. It is interesting to consider that, similarly to variations in the G1 and G2 phases (Calegari et al. 2005; Takahashi et al. 1995), S-phase length can be variable too. Lengthening of S-phase occurs during e.g. development (Duronio, Genes and Development, 2012, Nordman and Orr-Weaver, Development, 2012). The work by Arai et al. (2011) and Turrero García et al. (2016) report distinct lengths of S-phases according to distinct types of neural progenitors in the developing neocortex in mouse and ferret, respectively. In the adult mouse subependymal zone (Ponti et al., 2013), the *gfap*+ B-type cells displayed very rapid S-phases of

about 4-5 hours, which is below the values we measured here of about 18h by identifying double-labelled nuclei (see Figure 3F). Since we still detected occasional double labelled nuclei with 24h intervals, but not later, we can state with confidence that these nuclei were already in S-phase as the first label was present (until up to 4h after the injection) and finished their S-phase as the second labelled was administered. Hence, the group of NSCs defined by *gfap*:GFP+ in the zebrafish telencephalon undergoes collectively and comparatively long S-phases."

2. On page 11 the formula for f(x;beta,d) is incorrect. The function f is should be a probability density function of the cell cycle or S-phase length x. Yet, the formula presented is clearly not a probability density function. First, its integration in the range x in [0,+Infinity] does not yield 1. Second, it is inconsistent as it sums d, with dimensions time (e.g. hour), with a normalised exponential distribution that has no dimensions. I think the authors meant to write something like:

$$f(x;\beta,d)=\begin{cases}0 & x<0\\ \frac{1}{\beta}e^{-\frac{1}{\beta}(x-d)} & x\geq d\end{cases}$$

Alternatively, they may use a single equation with the Heaviside function as in the equation 1 of Weber et al (2014). Whether this was a simple typo or reflects the way the simulations were parameterised and performed in framework Morpheus must be re-examined by the authors.

We are grateful to the reviewer for pointing this out. We corrected the formula and fixed a typo ($x<d$ instead of $x<0$):

$$f(x;\beta,d)=\begin{cases}0 & x<d,\\ \frac{1}{\beta}e^{-\frac{1}{\beta}(x-d)} & x\geq d\end{cases}$$

3. The authors use a clever strategy to estimate the parameters of the their model of stochastic cell cycle entry and reentry based on intrinsic delayed-exponential distributed total cell cycle and S-phase lengths. They first estimate the four parameters of the two distributions ignoring all spatial constraints and then perform a spatial simulation using the framework Morpheus. This simulation has some additional ingredients, including the fixed division rate pdiv, the removal of "differentiated" cells after one division, the minimum and maximal cell size, and all the inherent parameters of the simulation. It was unclear how these additional ingredients affect final statistics of the cell cycle division in the simulations. What I mean is: did the authors reanalyse the results of the spatial simulations in the same way that they analysed the labelling data to make sure that effective cell cycle times are the same?

We thank the reviewer for this suggestion. We now show in Supplement Figure 4I-L (see also below) that we are able to reproduce the estimated observed re-division and DLS fractions as well as the cell cycle length. Also the internal re-division fraction was confirmed with 38+-0.5%. S-phase lengths are by default correct as we just draw them from the respective distribution for the post-simulation analysis. We describe this aspect in the revised version of our Results where we now write:

"Finally, we analyse whether the agent-based simulations are able to reproduce the input parameters, i.e. observed DLS and re-division fractions, cell cycle length and redivision probability. Re-division fractions and DLS fractions from simulations are similar to the ones observed in experimental data (Supplementary Figure 4I,J) ,and cell cycle lengths observed in the simulations fit nicely to the probability density function of the lag-exponential distribution (Supplementary Figure 4K). S-phase lengths are correct by default as we draw them after simulation for post-simulation analysis, from the respective lag-exponential distribution. The proportion of re-dividing cells in the simulation is confirmed with 38 ± 0.5 % (Supplementary Figure 4L)."

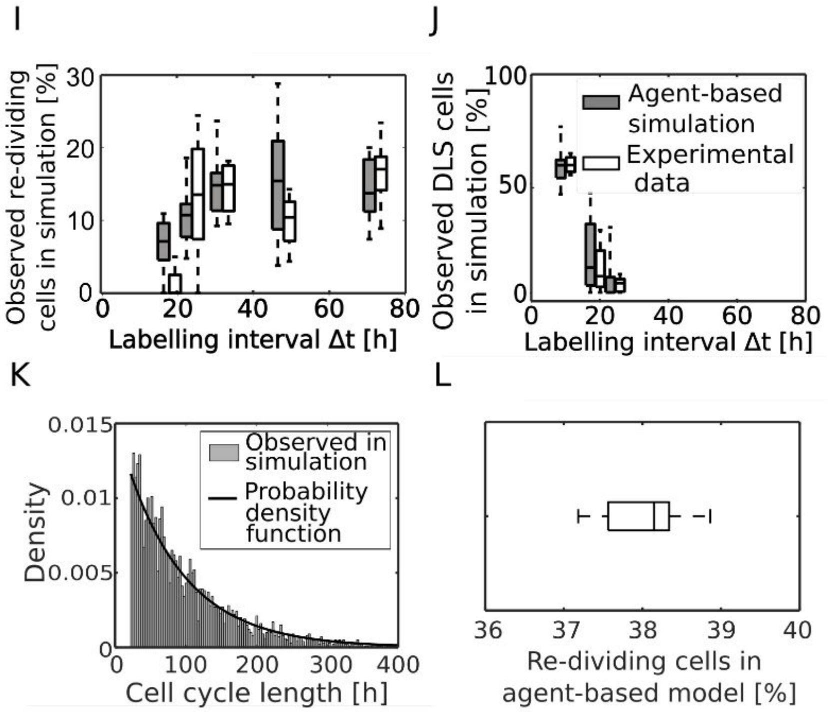

The additional ingredients namely the pdiv could in principle offset the effective cell cycle times in the simulations. There is nothing in the description of the simulations that ensures that this was not the case. Such biases could explain the difference in the interaction strength and radius.

We thank the reviewer for this remark. It is true that one cell has to wait at least one cell cycle duration until it is available for a spontaneous division via p_{div} but after cells are older than their cell cycle time there should be no impact anymore. We accordingly added a short paragraph in the method section where we now write:

“At the beginning of the simulation every cell has to wait at least one cell cycle duration until it is available for a spontaneous division via p_{div}. This leads to a small bias in the beginning (roughly 1/30th of the whole simulation time) but after all cells are older than their cell cycle time there should be no impact anymore.”

Additionally the estimated cell cycle length is based on times between division and the respective redivision as this is inferable from the observed data. As mentioned above, we show in the newly added Supplementary Figure 4K that effective cell cycle times in the agent-based simulation fit nicely to the probability density function of the lag-exponential distribution.

Other lesser issues:

4. The authors say that they detect “a non-random, aggregated patterns of dividing NSCs in the zebrafish brain.” The statement “non-random“ is inadequate and perpetuates the “common sense” notion that random equates to uniform probability (in this case uniform in space). The authors mean non-uniform or non-isotropic or .... The truth is they themselves later characterise the cell cycle and S-phase lengths, the cell-cycle reentry, the differentiation as random processes.

To motivate the used terminology, which we borrow from the book “Spatial Point Patterns: Methodology and Applications with R” from Baddeley et al., we inserted a Box on “Statistical analysis of spatio-temporal point patterns” in the main text:

Box: Statistical analysis of spatio-temporal point patterns

When events can happen at any point in a 2D Euclidean space, a **spatial Poisson process** leads to a point pattern that is completely defined by one parameter, the density of points. This **null model of complete spatial randomness** has been used for diverse analyses, from forest structures (Corral-Rivas et al., 2010) over accessibility of pediatric care (Tanimura and Shima, 2011) to road accident prevention (Nicholson, 1998). In our case, the space analyzed is not Euclidean, but **discrete**, since S-phase NSCs (the "events") only appear where NSC are already present in the zebrafish hemisphere. The corresponding null model is thus not a spatial Poisson process, but the distribution of randomly sampled NSCs.

Accordingly, we call an S-phase NSC pattern **random** if the distances between the events are not significantly different from randomly sampled S-phase NSCs. We evaluate this with an adapted version of a spatial statistics measure called **Ripley's K** (Ripley, 1976). **Non-random patterns** can be classified into two types: When events are closer to each other than expected from the random null model, we call the pattern **aggregated**. If events are further apart from each other, we call the pattern **dispersed**. A simulated random division pattern is displayed in Supplementary Figure 1B, a simulated dispersed division pattern on the same hemisphere is shown in Supplementary Figure 1D. The corresponding observed aggregated division pattern (with the same number of events) is shown in Figure 1E. We follow in our notation and nomenclature the book from Baddeley et al. (2015), which provides an overview and examples of diverse spatial statistics methods.

5. Page 4. "cells in S-phase straddling the Deltat and incorporating both labels". Does this mean that the cells spent more than Deltat in S-phase replicating their DNA? How can the authors distinguish if the cells where all the time in S-phase or were undergoing one S-phase during the first labelling and S-phase from a subsequent round of division during second labelling?

We thank the reviewer for this remark and now better explain the difference between DLS cells, which incorporates both markers within one cell cycle and re-dividing cells, which form a doublet (two small cells being close together):

"Short labelling intervals up to Δt=24h revealed cells in S-phase incorporating both labels (Figure 3A-F), hence denoted as double labelled S-phases (DLS). Remarkably, we also noticed cells with both S-phase markers that, unlike DLS, were arranged as doublets, i.e. two daughter cells close to each other. These cells apparently entered a second S-phase, appeared in labelling intervals of Δt=24h, 32h, 48h, and 72h (Figure 3G-T), and were denoted as re-divisions accordingly."

6. The authors' argument is one of parsimony. They argue that there is no need to evoke niches and supercellular interactions to explain the aggregation patterns because cell intrinsic rates of cell cycle entry and re-entry (re-division) would be sufficient to explain the patterns. Yet, this parsimony is more a modelling one than a biological one. What are the molecular mechanisms underpinning the rate of cell cycle re-entry or re-division? The authors make a fair discussion on the question of "how these recurrent divisions of adult stem cells come about". However, they may wish to acknowledge that it may be equally plausible mechanistically to explain the local reentry in cycle as a consequence of random foci of production of cell cycle progression signals (as mentioned in point 7 below).

Following the remark of Reviewer 4, which is similar to a comment from Reviewer 2, we extended the according paragraph on cell intrinsic and cell extrinsic mechanisms in the Discussion:

"According to this model, coordination of cell cycle entries in the population can be explained by the internal synchronization due to the cells' history. Such a model is supported by interesting studies following cell families in cultured cell lines, detecting correlations of cell cycle parameters between siblings and cousin cells as a result of inherited factors but independent on their location (Mura et al., 2019; Sandler et al., 2015).

This cell-intrinsic driven behavior is in contrast to cellular systems endowed with a clear environmental regulation, in particular in stem cell compartments constituted by a distinctive 3D architecture like in the bone marrow or the hair follicle in the skin. There, the precise location of stem cells is associated with their cycling behavior and fate (Rompolas et al., 2013), indicating that the position within an environment impacts on the stem cells activity. In the neurogenic zone studied here, no distinctive organization of the tissue would so far hint towards morphological features specifically associated with a NSC's activity. Our analysis of cellular volumes in this study does not indicate that a distinctive density dependent niche would be in place. In this context, it is also interesting to note that two intermingled types of progenitors with radial morphology in the killifish telencephalon reveal distinct proliferative behaviors (Coolen et al., 2020) even if sharing the same environment, arguing for a small contribution of environmental effects on stem cell activities.

Nevertheless, we cannot reject the existence of cell extrinsic mechanisms that might contribute to the spatio-temporal patterns, such as local diffusive signaling activity in delimited groups of cells, a functional activity of extended cell-cell contacts (as has been observed in NSCs by Obermann et al. (2019)), or the activity of the Notch signalling pathway (Chapouton et al., 2010). As a result of either mechanisms, levels of molecular heterogeneity have been observed: For instance, variable levels of the Zinc finger protein Fezf2 regulate Notch activity levels and quiescence of NSCs (Berberoglu et al., 2014). Likewise, the expression of miR9 involved in keeping quiescence upstream of Notch signaling is found only in a subset of quiescent cells (Katz et al., 2016) several days after a division. And in a recent study, a subpopulation of NSCs that expresses low levels of Elavl3 has been characterized as mostly non-dividing cells in transit towards neuronal differentiation (Lange et al., 2020)."

We also attempted to address the possible existence of niches around NSCs in division, where NSCs would display a distinct size. We did not measure any differences in the cell volumes. This result was added in our Supplementary Figure 3L-Q.

7. The modelling result that omitting intrinsic cell-cycle re-entry leads to random uniform spatial patterns is not general. It is specific to the simulations performed and based on the constant rate that the cells divide. Once the cells are placed according to a uniform distribution in space the constant rate results in a "random" spatial patterns. The lack of intrinsic re-division may not be incompatible with spatio-temporal aggregation pattern. Most likely if there would be transient foci of production of a growth factor necessary for overcoming G1 arrest (for example) one would see "random" cell division in space (as the foci would uniformly distributed in space) but with an apparent aggregation pattern and even apparent cell-cycle re-entry if the transient foci lasted long enough.

We agree with the reviewer that aggregated patterns could arise as the result of different mechanisms, including feedback loops and foci of activity as mentioned. We have extended our Discussion on the aspect of cell intrinsic versus cell extrinsic control of the activity of a stem cell population (see above). The fact that daughter cells re-enter division shows in itself a strong cell intrinsic component. However the patterns might arise by additional mechanisms. From a modeling perspective, following the argument of parsimony as the reviewer points out, we prefer the model with the least necessary assumptions, i.e. the re-division model. Here, foci appear naturally as the daughter cells of a dividing NSC have a higher chance to divide again.

8. The repeated use of the term "analogon" surprised me. I didn't recall reading or hearing it. I am used to "analog" or "analogue". Googling "analogon" brought mostly dictionaries and thesaurus related to German or Dutch. There are only 5 pages of papers using "analogon" in pubmed versus, about 5000 using "analog" or "analogue". These precedents of using "analogon" notwithstanding, maybe the authors should consider adopting the more conventional term.

Thanks for the remark, we changed the term to "analog".

9. Section "A positive interaction model fits..." (page 4). It would help the reader if the authors would explain the rational and interpretation of the "interaction strength". The reference to Lupperergerger et al. and the formal definition in the corresponding methods section is not sufficient to allow the reader to make sense of "we find that a model with an interaction radius of ~ 100 µm and an interaction strength > 1 describes the data best". See also the next point.

We revised the section accordingly and now try to better explain interaction strength by writing:

"[...] we use the temporal extension of a spatial model (Lupperger et al., 2018) that allows inferring the most likely parameters for interaction strength and interaction radius for an arbitrary number of datasets. Aggregated patterns emerge for an interaction strength > 1, random patterns for strength = 1, and dispersed patterns for strength < 1 (see Methods)."

10. Fig. 3B. The interaction strength and radius seem not to be independent of each other, showing a conspicuous negative correlation. Why is that? How does it affect the result? Furthermore, the posterior distribution seems to be multimodal with some periodicity as a function of the radius. This might be an optical illusion but similar patterns appear in supplementary fig. 3. Is this an effect of the ABC sampling? Is the most "most likely radius" of 93µm the point of higher probability density?

We thank the reviewer for this interesting remark. However, the two parameters are not per se coupled. We can easily simulate patterns where strength and radius are decoupled (see Figure below, upper row) or show other forms of correlations (lower row). In our data correlations most probably appear because the observed patterns can be explained best with particular strength / radius combination.

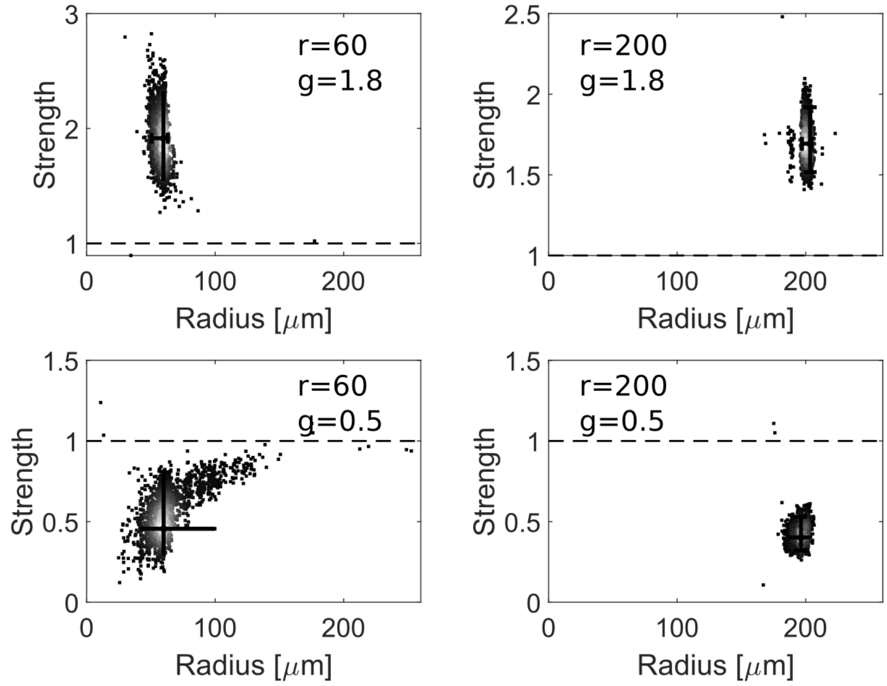

Concerning the mentioned multimodality we indeed sometimes (especially in 9h experiments) see more than one mode. This is most likely the consequence of the multi hemisphere input to the model. One mode would correspond to the preferred parameter combination of one hemisphere, while the other hemispheres might prefer another optimal parameter combination. The ABC approach itself has no effect on multimodality in the posterior sampling as it is part of the cell division model while strength and radius estimation are part of the interaction model.

We updated Figure 3B (now Figure 2B) and now plot the density of the posterior sampling to show that the most likely radius is indeed in the middle of the highest density region.

11. Page 10. "We also observed re-divisions in gfap:GFP- cells, which were clearly distinguishable from each gfap:GFP+ cells (Supplementary Figure 4A) and occured in similar proportions (Supplementary Figure 4J)." This sentence is confusing and should be better explained. Does this mean that NSC (GFP+) and other cells (GFP) divide with the same temporal patterns?

We thank the reviewer for pointing this out. We tried to clarify our message and now write:

"We are confident that we are observing this phenomenon in stem cells, as we could clearly distinguish between the events taking place in *gfap*:GFP+ and *gfap*:GFP- progenitors (Supplementaries Figure 3A and Table 2). Reoccuring divisions in NSCs happened in similar proportions as in the *gfap*:GFP- progenitors, the latter being considered so far as transit amplifying progenitors (Supplementary Figure 3B,C)."

12. The authors report the uncertainty of their data with a 2 significant figure e.g. 14.4±8.1% on page 10. Although reporting of data is increasingly relaxed in the scientific literature it is worth recalling the rule that the reported estimated values should be rounded to the first decimal of the uncertainty.

Thanks for the remark. We now write: "14 ± 8%"

13. Page 10, second paragraph starting with "To confirm our observations,...". Are the results consistent with a stationary process and balanced growth of the tissue ? Are the statistics on the first (L1) to second (L2) labelling the same as for the second to third (L3) labelling? What I envisage is a 4 by two contingency table of the type: rows L1-L2-, L1+L2-,L1-L2+,L1+L2+ vs row first set L1L2:BrdU-24h-EdU and second set L1L2:EdU-24h-PCNA. Are the values in these tables the same? The similarity could be addressed using Chi2 test.

We thank the reviewer for raising this important point. The question of a stationary process is difficult to answer here, because we identify considerably more PCNA+ cells due to its expression throughout the full cell cycle as compared to BrdU and EdU which label only S-phases. Thus, the first to second labeling are difficult to compare. However, for completeness we now provide the contingency tables in Supplementary Table 3 and below. As suggested, we analyzed (BrdU48h/Edu24h vs. Edu24h/PCNA) using a chi squared test. The resulting p-values are significant for Brain2R and Brain1R+1L (p-values of 0.003 and 0.0003 respectively) while the p-value of Brain2L (p-value of 0.02) is borderline and does not allow to reject the null hypotheses of both rows being associated. However, this set of experiments still shows that the use of a third cell cycle label also reveals cell cycle re-entries of NSCs, as in the first set of experiments.

We show the results in a pie chart displaying the proportions of dividing NSCs that have been labelled by a previous label (Supplementary Figure 3K), in addition to the Supplementary Tables 3 (see below).

Category	Brain1	Brain2L	Brain2R	Brain1 %	Brain2L %	Brain2R %	Average
BrdU+ only	9	13	16	1,56%	1,56%	1,66%	1,60%
EdU+only	2	11	4	0,35%	1,32%	0,42%	0,69%
PCNA+only	25	27	29	4,34%	3,24%	3,02%	3,53%
BrdU+EdU+	4	4	5	0,69%	0,48%	0,52%	0,56%
EdU+PCNA+	8	14	16	1,39%	1,68%	1,66%	1,58%
BrdU+PCNA+	8	10	5	1,39%	1,20%	0,52%	1,04%
no marker	520	755	886	90,28%	90,53%	92,20%	91,00%
Total	576	834	961	100%	100%	100%	100%

	Brain1	Brain2L	Brain2R	Average	Stdev
%PCNA+ previously dividing	39,0%	47,1%	42,0%	42,7%	4,1%
% EdU+ previously dividing	28,6%	13,8%	20,0%	20,8%	7,4%

Brain2R	L1-L2-	L1+ L2-	L1-L2+	L1+L2+	Total
BrdU48h/EdU24h	915	21	20	5	961
EdU24h/PCNA	902	9	34	16	961

Brain2L	L1-L2-	L1+ L2-	L1-L2+	L1+L2+	Total
BrdU48h/EdU24h	782	23	25	4	834
EdU24h/PCNA	768	15	37	14	834

Brain 1L+1R	L1-L2-	L1+ L2-	L1-L2+	L1+L2+	Total
BrdU48h/EdU24h	545	17	10	4	576
EdU24h/PCNA	529	6	33	8	576

14. Page 6. The authors use incorrectly the term “predicted” to refer to “estimated” values, as in the sentence “It predicted a minimal cell cycle time of 22.2h”.

Thanks! We changed the term and now write:

“It estimated a minimal cell cycle time of 22.2h [...].”

15. The authors denoted by beta the mean of the exponential component of the delayed-exponential cell cycle length distribution. This is a different notation from the one used in the reference they cite (Weber et al., 2014). In Weber et al. alpha and beta are respectively the mean of the exponential and the delay of the delayed- exponential. There is nothing wrong with adopting a new notation (beyond the faulty equation mentioned above). To avoid confusing the reader, however, may be the authors should make a brief note about this.

We thank the reviewer for spotting this inconsistency. However, we would like to keep our notation as β is commonly known as the exponential scale parameter (see e.g. https://en.wikipedia.org/wiki/Exponential_distribution). Following the reviewer’s suggestion, we added a brief note in the Methods where we write:

“[...] where β is the scale parameter of the exponential distribution and d is the delay. Note that (Weber et al., 2014) use a different notation for the two parameters.”

16. The authors estimated a probability of cell cycle re-entry of 0.38, which is, on face value, not very different from 0.5. Is the value 0.5 within the confidence or credibility interval of the estimate? Would the results be very different if the authors would impose a value of 0.5? Intriguingly, a value of 0.5 could be interpreted as asymmetric cell division which is one of the classical models for stem cell self-renewal and differentiation. Maybe the authors would like to address this issue.

The reviewer raises an interesting point. We inspected the parameter estimates from our final ABC epoch and found that 0.5 is outside the 5-95% range of the re-division probability (see Figure below). However, we would like to note that the redivision rate is independent from division symmetries. That is in our model, each daughter cell will undergo another division with probability 0.38, independent from the other daughter and without any impact on the division outcome, i.e. remaining stem cell fate or differentiation.

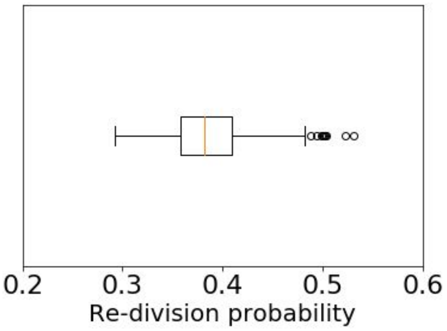

17. Maybe the authors would like to discuss their results in the context of the article by Mura et al. (2019; doi: 10.1371/journal.pcbi.1007054).

We thank the reviewer for pointing to this interesting study, which indeed revealed to us new aspects on the impact of cells' history on the distribution of behaviors in a cell population. We now cite this paper, as well as Sandler et al. (Nature, 2015) in our revised Discussion.
