# Supplementary material for: Reoccurring neural stem cell divisions in the adult zebrafish telencephalon are sufficient for the emergence of aggregated spatiotemporal patterns
Source: PLoS Biol. 2020 Dec 8;18(12):e3000708. doi: 10.1371/journal.pbio.3000708 (PMC7748264; doi:10.1371/journal.pbio.3000708)
Supplement: Attachment — Submitted filename: Response.pdf [file pbio.3000708.s009.pdf]

Reviewer #3: I consider that the authors addressed all my previous points. After reading the new version of the manuscript I find it significantly improved. I am in favor of publishing the manuscript after the following points are being addressed:

1) On the page 7, last subsection of the Results section, the authors wrote "We now fed an agent-based model, implemented in the Morpheus toolbox". This sentence omits the modelling class that the model has, which is definitely more important than the software that the authors used to implement the model. Along the manuscript there is no reference to the fact that the authors use a Cellular Potts Model. This modelling class should be acknowledged in the text of the manuscript, together with an argument of why they favored this modelling class. In fact, they could have chosen Cellular Automata or Vertex model, among others. But CPM has some advantages that the authors used and some disadvantages that the authors circumvented. Hence, it is important to at least briefly elaborate on this.

We agree that the modelling class is an important point to discuss. We thus revised the Results, writing

“To model cell shape kinetics, cellular potts models (Graner and Glazier 1992), vertex models (Alt et al. 2017) and cellular automata (Manukyan et al. 2017) have been used in the past. We chose a cellular potts model, since it allows for arbitrary shapes and stochasticity in cell movement, and is easily applicable as the default implementation in the Morpheus environment (Starruß et al., 2014).”

and the Methods, where we now write:

“To assess the contribution of re-division events to the emerging spatial patterns of NSCs in S-phase, we simulated an NSC population using a cellular Potts model as implemented in Morpheus (Starruß et al., 2014).”

2) In the Supplementary Figure 4B, the authors show that cells divide immediately after S-phase. Nevertheless, this implies that G2-phase is nonexistent. Why is that? Did the authors have an estimation of the length of this phase and know that it is negligible compared to the others? If this is the case, this should be acknowledged in the manuscript.

Following the reviewer’s point, we revised the caption of Supplement Figure 4, where we now write:

“Note that we do not have any information about the length of G2 and thus do neither model nor visualize this phase in the graphs.”

3) The authors experimentally detected that the probability of re-division was ~.15. Yet, the fitting of the simple model after the ABC fitting returned a probability of ~0.38. What is the posterior distribution of this parameter? The authors mentioned in the methods section, subsection "Cell division model" that they used .15 as a lower boundary. Thus, the experimental value and the model estimate are not significantly different from each other? If the authors fix this parameter in 0.15 in the model and repeat the ABC fitting, would they obtain similar results? The authors mentioned in the legend of Supp. Fig. 4 B this difference, but I think this point should be acknowledged in the results section. The authors should at least discuss why there is this discrepancy.

We thank the reviewer for raising this important point. In the revised Results section of the manuscript, we now state clearer that these are two different parameters by writing::

“Note that while the re-division probability is a parameter of our model, the re-division fraction is an experimental observable, which depends on the measurement method. Using snapshot measurements, we found a re-division fraction of 15% re-divisions, which is considerably lower than the re-division probability of 38% (see S4B Fig for a detailed explanation).”

Regarding the question about fixing the re-division parameter to 0.15: We simulated patterns with various re-division probabilities. We found that already for a re-division probability of 0.20 aggregated pattern can no longer be significantly detected.

4) In Legend of Fig. 4 A, there is a grammar problem. Please re-write. Also, what is the meaning of grey and black in Fig. 4 C, D, G and H?

We revised the caption to Fig 4A where we now write:

“We use an agent-based cellular Potts model model to simulate NSC divisions with a re-division probability of $p_{re\text{-}div}$=0.38 and perform virtual measurements with labelling intervals Δt between 9h and 72h (here shown for Δt = 48h).“

We added the missing information about the black and gray coloring by writing:

“Labelling intervals that are also available from experimental data (see Fig 2C) are shown in black, all others in gray.”

PLOS Formatting Requests:

MANUSCRIPT REQUIREMENTS

Please update your manuscript with the following changes:

1. Please upload your main article file as a .doc, .docx or .rtf file.

Main article uploaded as docx.

2. In your Data Availability Statement, please ensure you have provided all necessary access information in complete sentences. This statement will appear on the published version of your manuscript.

The data availability statement provides all information to access the data in complete sentences.

3. Please ensure that all authors have at least one affiliation listed on the title page and that all listed affiliations are attributed to at least one author. Affiliations should be listed in ascending numerical order in the affiliation list and should match what is provided in the Authors Information in the submission form.

Affiliations should include an institution, a city, and a country (no postcode). US affiliations need the state as well, written out in full (with no abbreviations).

We updated the affiliations.

4. **Please ensure that all author names are spelled correctly in the manuscript file and the submission form, including the use of a middle name/initials, and any special characters (accents, umlauts, etc.). Please place first/given names before last/family names. We noticed that you only use first initials on the title page for each of your authors, while you use full first names on the submission form, and ask that you ensure they match verbatim. Please be aware that correct spelling and formatting of author names in the article file is vital for accurate indexing in PubMed.**

Changed to full first names.

5. We have noted that you have the following present in your manuscript: Author Contributions/ Data Availability Statement

Statements deleted.

We will remove these section from your manuscript file as this information is automatically pulled from the submission form. Please make sure that the information in the submission form is correct.

Submission information updated.

6. Please ensure all equations are inserted in MathType or Word Equation Tool. You can download a free trial of MathType here: http://www.dessci.com/en/products/mathtype/.

Equations inserted in MathType.

7. **Please remove the funding information from the Acknowledgments section of your manuscript file. The published version is pulled automatically from the "Financial Disclosure" field in our submission form and must not be duplicated. Please therefore take this opportunity to check that the information in this field is correct and completed in full.**

Section removed.

8. **Please cite boxes in ascending numerical order in your main article file.**

Boxes are cited in ascending numerical order.

9. **Please make sure all references are cited in ascending numerical order in the text.**

Checked.

10. **Please upload your Supporting Information items individually, rather than in a single file, as you refer to them individually in the text. If you would like to retain the single file please rename internal items (e.g. tables) as A, B, C etc. Please also provide a legend for each under the heading Supporting Information. Supporting Information files should be less than 10 MB each. Please edit any citation of internal items within your Supporting Information files to using letters rather than numbers, e.g. "Table A in S1 Text". Please ensure these changes are made throughout the manuscript and within the Supporting Information files themselves.**

Supporting information is now uploaded in separate files and are adapted.

11. Please ensure that all Supporting Information files use the following format verbatim throughout the manuscript (including legends and all in-text citations): S1 Fig, S1 Table, S1 Text, S1 Data, etc. Each file type should start at 1 and increase numerically. Please note, however, that the file names themselves must use an underscore rather than a space, i.e. S1_Fig.tif, S1_Table.xlsx, S1_Text.doc, S1_Data.xlsx, S1_PRISMA_Checklist.docx, etc.

Citations changed to the right format.

12. Please ensure that each supporting information file has a legend at the end of the manuscript file, after the Reference list.

Legends added.

13. Please note that your submission cannot contain any references to generic "supporting information" files and instead must refer to specific files using our naming convention (S1 Fig, S1 Table, S1 Text, S1 Data, etc.)

We now only include specific supporting references.

Before resubmitting your paper, please ensure that all files referenced in the manuscript are present in the File Inventory, as missing files will result in further delays. Please also ensure that all file names adhere to our naming conventions and that all in-text citations and supporting information file legends/captions do as well.

If Supporting Information files are included with your article, note that these are not copyedited and will be published as they are submitted. Please ensure that these files are legible and of high quality (at least 300 dpi) in an easily accessible file format. For this reason, please be aware that any references listed in an SI file will not be indexed. For more information, see our Supporting Information guidelines.

Checked and adapted.

FIGURE REQUIREMENTS

1. Please ensure that all main figures are referenced in the manuscript as Fig 1, Fig 2, etc. (including capitalization), rather than Figure 1, Figure 2, etc. Please note, however, that the file names themselves must not include the space, i.e. Fig1.tif, Fig2.tif. Please ensure that all main figure files are clearly labeled as such in the File Inventory.

Figure references updated.

2. Please note that your figures do not currently comply with our publication requirements. For further instructions to assist with your Figure revisions, please refer to our Figure Guidelines at: http://journals.plos.org/plosbiology/s/figures.

If you need assistance, please contact us directly at graphics@jjeditorial.com so we can further help with this matter. Please send any relevant source files and tell us what application(s) and what operating system you are using.

- Please reformat Figures 1 and 3 to have a white background with no border.

Figures changed and changed figures were checked via PACE and approved by graphics@jjeditorial.com.

- Figures 1 and 3 look like they were originally at a lower resolution and will appear blurry when published. This error cannot be fixed by simply increasing the resolution of your figures with image editing software. Instead, please carefully review the PLOS Figure Guidelines and provide figure files that were originally created at a minimum of 300 pixels/inch (ppi) and that are sized between 6.68-19.05 cm wide and no more than 22.225 cm tall (excluding any white space around your artwork). Additionally, please ensure your figures are in RGB colour mode.

Figures uploaded with higher quality.

- Panel A of Figure 2 and some lines in graphs in panels B and F of Figure 4 appear pixelated/low quality. Please remake these parts of the figures using the original source, using the PLOS figure guidelines to ensure quality is preserved [http://journals.plos.org/plosbiology/s/figures].

Figures uploaded with higher quality.
